# Whole-Exome Sequencing Explores Host Genetic Susceptibility of COVID-19 Severity in Unvaccinated Vietnamese Patients Infected with the Delta Variant: A Hypothesis-Generating Study

**DOI:** 10.3390/genes17080959

**Published:** 2026-08-15

**Authors:** Hoai Thu Thi Nguyen, Thanh-Van Ta, Thuy Thi Le, Quyen-Diep Nguyen, Van Cao, Chan Dinh Khac Nguyen, Tuan Nguyen Duc, Ngoc-Lan Thi Nguyen

**Affiliations:** 1 Department of Biochemistry, Hanoi Medical University, Hanoi 100000, Vietnamtathanhvan@hmu.edu.vn (T.-V.T.); diepquyen@hmu.edu.vn (Q.-D.N.); ductuannguyen@hmu.edu.vn (T.N.D.); 2 Faculty of Medical Laboratory Science, Danang University of Medical Technology and Pharmacy, Da Nang 550000, Vietnamvan.cao@dhktyduocdn.edu.vn (V.C.); 3 Biomedical Research and Application Center, Phan Chau Trinh University, Da Nang 550000, Vietnam; cdn14@georgetown.edu; 4 Department of Clinical Laboratory, Hanoi Medical University Hospital, Hanoi 100000, Vietnam

**Keywords:** COVID-19, whole-exome sequencing, host genetics, gene-based association, systems genetics, functional genomics, RNA processing, mitochondrial function, network biology, Vietnamese population

## Abstract

**Background/Objectives**: Host genetic variation contributes to the heterogeneous clinical outcomes of coronavirus disease 2019 (COVID-19), yet evidence from Southeast Asian populations remains limited. **Methods**: We investigated host genetic factors associated with COVID-19 severity in Vietnamese patients infected with the SARS-CoV-2 Delta variant using whole-exome sequencing. A total of 48 unvaccinated patients were enrolled. During sample-level quality control, one sample was excluded because the genetically inferred sex was discordant with the information recorded in the clinical medical record, leaving 47 samples (23 severe/critical and 24 mild/asymptomatic) for downstream genetic analyses. Gene-based association analysis was performed using MAGMA, followed by functional enrichment and interaction network analyses with Metascape and GeneMANIA. **Results**: Population structure analysis showed that the study participants clustered closely with the East Asian (EAS) reference population. No individual variant reached the prespecified multiple-testing-adjusted threshold, and no gene-level association survived Benjamini–Hochberg correction. Using a nominal MAGMA gene-based *p* < 0.01 threshold solely for exploratory prioritization, 44 genes were selected for downstream functional analyses. Functional enrichment highlighted RNA processing and mRNA maturation, together with mitochondrial, metabolic, immune-regulatory, and cellular homeostasis pathways. Network topology analysis further identified several highly connected genes, including CPSF4, MRPS34, ATP5MF, BUD31, SNRPD3, and HDAC1. **Conclusions**: These hypothesis-generating findings are consistent with a potentially polygenic contribution to severe COVID-19 and provide an exploratory systems-level framework for understanding host genetic susceptibility in the Vietnamese population, while identifying biologically plausible candidate genes and pathways for future validation.

## 1. Introduction

Coronavirus disease 2019 (COVID-19), caused by severe acute respiratory syndrome coronavirus 2 (SARS-CoV-2), remains a major global public health challenge because of its substantial morbidity, mortality, and socioeconomic impact. Although most infected individuals develop asymptomatic or mild disease, approximately 15–20% progress to severe pneumonia requiring hospitalization, while a smaller proportion develop acute respiratory distress syndrome (ARDS), multiorgan failure, or death. Advanced age, male sex, obesity, diabetes mellitus, cardiovascular disease, and chronic respiratory diseases are well-established risk factors for severe COVID-19. However, these factors alone do not fully explain the marked interindividual variability in clinical outcomes, as severe disease may also occur in young individuals without underlying illnesses, whereas some elderly patients with multiple comorbidities recover uneventfully. These observations suggest that inherited host genetic variation contributes to individual susceptibility and disease severity following SARS-CoV-2 infection [1,2,3,4].

Since the beginning of the pandemic, extensive efforts have been undertaken to identify host candidate genes associated with COVID-19 severity. Large-scale genome-wide association studies (GWASs), particularly those conducted by the COVID-19 Host Genetics Initiative, have identified multiple susceptibility loci involved in antiviral immunity, inflammatory responses, pulmonary epithelial biology, and coagulation pathways. Among the most consistently replicated loci are variants in or near LZTFL1, IFNAR2, OAS1/OAS2/OAS3, TYK2, DPP9, and the ABO locus, highlighting the importance of type I interferon signaling, innate antiviral defense, and dysregulated inflammatory responses in severe COVID-19. Collectively, these studies have established host genetics as an important determinant of COVID-19 outcomes and substantially advanced our understanding of disease pathogenesis [5,6,7,8,9].

Despite these advances, important limitations remain. Conventional GWASs are primarily designed to detect common variants with relatively small effect sizes and frequently identify association signals within non-coding regions, making biological interpretation challenging. In addition, rare and low-frequency coding variants, which may exert larger functional effects on protein structure and host immune responses, are generally underrepresented in GWAS datasets. Consequently, complementary sequencing-based approaches are required to identify functional coding variants that may directly influence host responses to SARS-CoV-2 infection [8,9,10,11].

Whole-exome sequencing (WES) has emerged as a powerful approach for investigating rare coding variants because it directly interrogates protein-coding regions, where a substantial proportion of disease-causing variants are located. During the COVID-19 pandemic, WES studies have identified rare deleterious variants affecting genes involved in type I interferon immunity, innate antiviral defense, cytokine regulation, lymphocyte activation, and inflammatory signaling in patients with life-threatening disease. Compared with conventional GWASs, WES provides greater biological interpretability by linking genetic variation directly to protein-coding genes and has therefore become an important complementary strategy for elucidating the molecular basis of severe COVID-19 [10,11,12,13].

However, severe COVID-19 is unlikely to result from dysfunction of a single susceptibility gene. Rather, disease severity probably reflects the cumulative effects of multiple genetic variants acting within interconnected biological pathways and molecular networks. Consequently, recent genomic studies have increasingly adopted gene-based association methods and systems biology approaches to move beyond single-variant discovery toward identifying coordinated biological processes underlying disease phenotypes. Gene-based analytical methods, such as Multi-marker Analysis of GenoMic Annotation (MAGMA), increase statistical power by aggregating association signals across variants within individual genes, while downstream functional enrichment and network analyses facilitate biological interpretation by placing candidate genes into their functional context [14,15,16,17,18].

Despite substantial progress in COVID-19 host genetics, important knowledge gaps remain, particularly in Southeast Asia. Most genomic studies have been conducted in European populations or genetically heterogeneous international cohorts. Differences in allele frequencies, linkage disequilibrium structure, population genetic architecture, circulating viral variants, vaccination status, and previous SARS-CoV-2 exposure may all influence genetic association results, thereby limiting their generalizability across populations [5,6,8]. Population-specific genomic studies are therefore essential for identifying both shared and ancestry-specific candidate genes of COVID-19 severity.

Whole-exome sequencing studies in Vietnamese patients with COVID-19 remain particularly limited. To our knowledge, only one published WES study has investigated host genetic susceptibility to COVID-19 in a Vietnamese population, providing the first evidence that host genetic variation may contribute to disease severity [13]. Nevertheless, interpretation of exome-wide findings remains incomplete because gene-based association analyses and integrative systems biology approaches were not comprehensively applied. Consequently, the functional relationships among candidate genes and the biological pathways contributing to severe COVID-19 in Vietnamese patients remain poorly characterized.

Vietnam experienced several major waves of the COVID-19 pandemic, with the Delta variant accounting for most severe cases before widespread vaccine coverage. In the present study, all enrolled patients were infected during the Delta wave, had laboratory-confirmed SARS-CoV-2 infection by real-time reverse transcription polymerase chain reaction (RT-PCR), had not received any COVID-19 vaccine before infection, had no documented history of previous SARS-CoV-2 infection, and had no known pre-existing immunodeficiency or immunosuppressive therapy. Compared with cohorts comprising multiple viral variants, heterogeneous vaccination status, or repeated infections, this biologically homogeneous cohort reduces several important epidemiological confounders and facilitates a more focused exploratory evaluation associated with disease severity.

Building upon previous WES studies, the present investigation was designed not only to identify candidate genes associated with severe COVID-19 but also to explore the biological processes through which these genes may collectively contribute to disease progression. We therefore performed whole-exome sequencing followed by gene-based association analysis using MAGMA and complementary systems biology analyses, including Gene Ontology (GO), Kyoto Encyclopedia of Genes and Genomes (KEGG), Reactome, Hallmark, Immunologic Signature, and GeneMANIA network analyses. By integrating gene-based association testing with multiple functional analytical approaches, this exploratory study aimed to provide a systems-level framework for interpreting potential biological processes associated with severe COVID-19 in Vietnamese patients and to prioritize biologically plausible candidate genes and pathways for future validation.

## 2. Materials and Methods

### 2.1. Study Design and Participants

This exploratory cross-sectional whole-exome sequencing (WES) study adopted a hypothesis-generating systems genetics approach to identify host genetic factors and biological pathways associated with COVID-19 severity. Participant recruitment was conducted at multiple COVID-19 treatment centers across Vietnam between May 2021 and October 2021, corresponding to the period when the SARS-CoV-2 Delta variant was the predominant circulating lineage in the country. For patients hospitalized prior to the formal Institutional Review Board (IRB) approval date (7 July 2021), genomic DNA was retrospectively extracted from residual blood samples initially collected for routine clinical laboratory testing, in strict accordance with the IRB-approved retrospective inclusion protocol. From July 2021 onwards, prospective recruitment and sample collection were performed. DNA extraction, whole-exome sequencing, and downstream bioinformatic and statistical genetics analyses were performed at Hanoi Medical University.

A total of 48 unrelated, unvaccinated (vaccine-naive prior to infection) Vietnamese patients with laboratory-confirmed SARS-CoV-2 infection were enrolled. According to the COVID-19 treatment guidelines of the Vietnamese Ministry of Health, participants were classified into two clinical groups:

- Mild/asymptomatic group (*n* = 24): Patients presenting with mild symptoms or remaining asymptomatic.
- Severe/critical group (*n* = 24): Patients who progressed to severe or critical COVID-19, presenting without major underlying comorbidities at the time of hospital admission.

Disease severity was determined based on the most severe clinical status documented during hospitalization. All patients had RT-PCR-confirmed SARS-CoV-2 infection during the Delta variant wave. Key exclusion criteria included a history of prior COVID-19 infection, pre-existing immunodeficiency, or treatment with immunosuppressive therapies before infection. The primary outcome variable was COVID-19 severity, dichotomized into mild/asymptomatic versus severe/critical disease. Clinical demographic and comorbidity information, including age and sex, was obtained from the medical records of the enrolled participants and was summarized for the clinical cohort (*n* = 48). For the downstream genetic association analyses, only participants who passed the predefined sample-level and variant-level quality-control procedures were retained. Age, sex, and ethnicity from the records, together with the first three principal components derived from genome-wide genetic data, were included as covariates in the genetic association models to account for potential clinical confounding and population stratification.

### 2.2. Clinical Data Collection, Sample Collection, and Genomic DNA Extraction

Clinical and demographic information, including age, sex, and documented comorbidities, was obtained from the available medical records of all 48 enrolled participants. The available records contained limited clinical information beyond basic demographic characteristics and selected documented comorbidities. These variables were used to characterize the clinical cohort and are summarized in Supplementary Table S1. Detailed anthropometric measurements, including height and weight required for BMI calculation, smoking status, and comprehensive biochemical or laboratory parameters were not consistently available. Information regarding specific inpatient therapeutic regimens was also incomplete. Therefore, these variables could not be incorporated into the genetic association models.

Peripheral venous blood was collected for genomic DNA extraction. Genomic DNA was isolated from whole blood using the QIAamp DNA Mini Kit (QIAGEN, Hilden, Germany) according to the manufacturer’s instructions. DNA quantity and purity were assessed prior to library preparation, and samples meeting the manufacturer’s quality requirements were stored at −20 °C until sequencing analysis.

### 2.3. Whole-Exome Library Preparation and Sequencing

Whole-exome libraries were prepared using the Illumina DNA Prep with Exome 2.5 Enrichment Kit (Illumina, San Diego, CA, USA) according to the manufacturer’s instructions. Following library quality assessment, paired-end sequencing was performed on the Illumina NextSeq 1000/2000 platform, generating raw sequencing data in FASTQ format for downstream bioinformatic analyses [19,20].

### 2.4. Overall Bioinformatics Workflow

**Figure 1:**
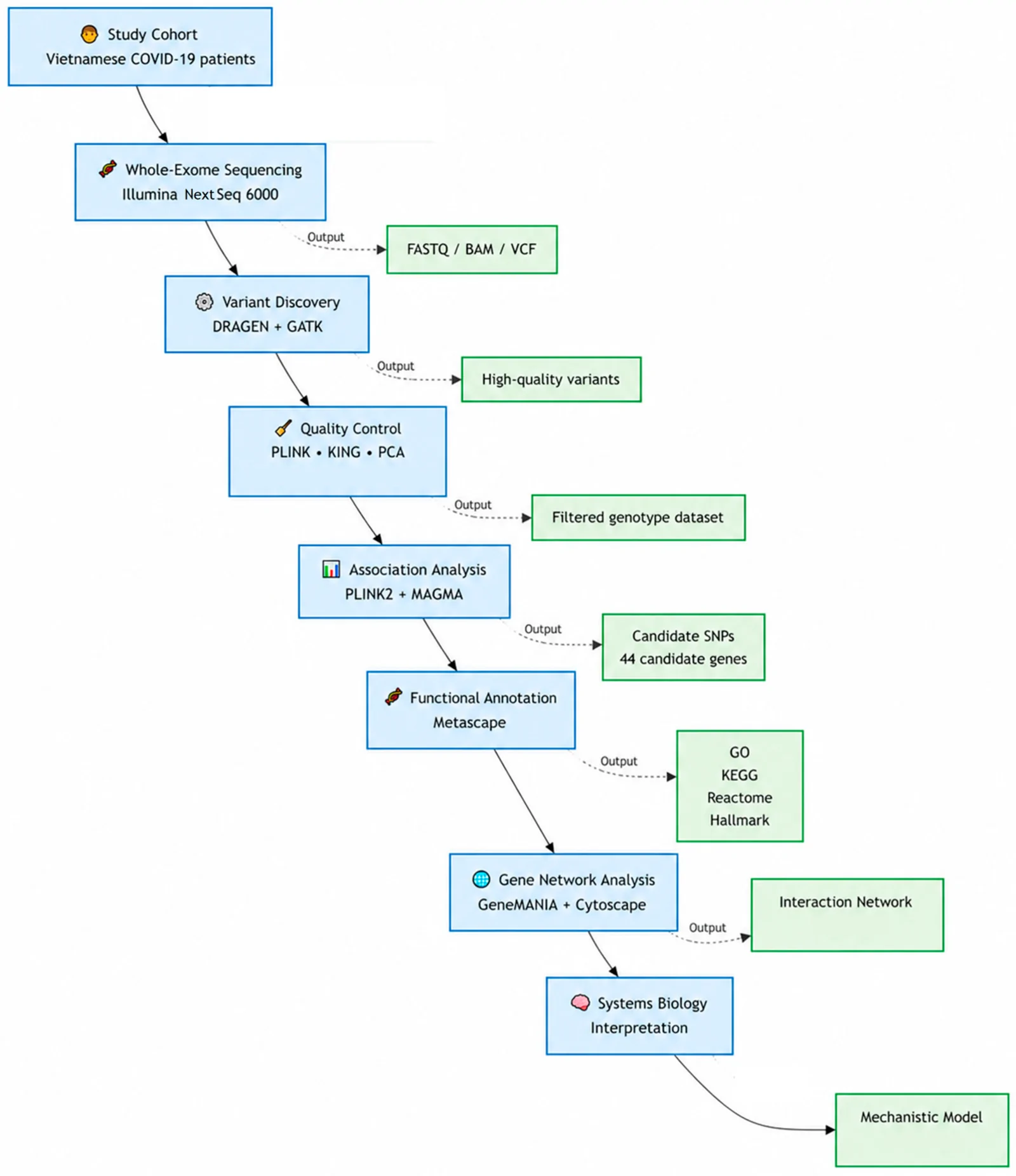
Overall analytical workflow of the study. Whole-exome sequencing data from 48 Vietnamese patients with mild/asymptomatic or severe/critical COVID-19 were processed through variant discovery, quality control, association analyses, functional analyses, and systems biology modeling. The analytical pipeline generated candidate genes for downstream enrichment, network, and mechanistic analyses.

The complete analytical workflow is illustrated in Figure 1. Single-variant association analysis was performed using PLINK2, whereas gene-based association analysis was conducted using MAGMA. Functional enrichment analyses were carried out using Metascape, followed by gene interaction network analysis using GeneMANIA and network visualization in Cytoscape. Finally, an integrative systems biology model was developed to summarize the potential molecular mechanisms associated with COVID-19 severity.

### 2.5. Bioinformatics Processing and Variant Calling

Raw sequencing reads in FASTQ format were processed using the Illumina DRAGEN Germline Pipeline with the GRCh38/hg38 human reference genome. The pipeline included read alignment, duplicate marking, base quality score recalibration, and germline variant calling to generate genomic variant call format (GVCF) files for each sample. The DRAGEN Germline Pipeline has demonstrated analytical performance comparable to conventional GATK-based workflows. Individual GVCF files were merged using GATK CombinGVCFs, followed by joint genotyping with GATK GenotypeGVCFs to generate a multisample variant dataset. Variant normalization was performed using bcftools norm, and variant quality was refined using the GATK Variant Quality Score Recalibration (VQSR) workflow. The final high-confidence variant dataset was exported in Variant Call Format (VCF) and converted into PLINK binary format for downstream quality control and genetic association analyses [21,22].

### 2.6. Genotype Quality Control

Genotype quality control (QC) was performed using PLINK following standard procedures for genome-wide genetic association studies. QC was conducted at both the sample and variant levels before downstream analyses. Samples with genotype missingness > 5% were excluded. Concordance between the sex recorded in the clinical medical records and genetically inferred sex was assessed using X-chromosome heterozygosity. One sample showed discordance between the reported clinical information and the genetically inferred sex and was excluded from subsequent genetic analyses. Sample heterozygosity and contamination metrics were also assessed, and pairwise relatedness was evaluated using KING; only unrelated individuals were retained for downstream analyses.

At the variant level, SNPs with genotype missingness > 5%, minor allele frequency (MAF) < 1%, or Hardy–Weinberg equilibrium (HWE) *p* < 1 × 10^−5^ were excluded. Linkage disequilibrium (LD) pruning was performed before principal component analysis (PCA), and genomic regions with extended LD, including the human leukocyte antigen (HLA) region, were excluded. PCA was subsequently performed using the 1000 Genomes Project reference panel to characterize population genetic structure. The first three principal components were subsequently included as covariates in the genetic association models to account for potential population stratification [23].

### 2.7. Single-Variant and Gene-Based Association Analyses

Following quality control, variants with a minor allele frequency (MAF) ≥ 1% were retained for downstream genetic association testing. Genome-wide single-variant association analyses were performed in PLINK 2 using additive logistic regression, with COVID-19 severity as the binary outcome. The regression models included age and sex obtained from the clinical records, together with the first three principal components (PC1–PC3) derived from genome-wide genetic data, as covariates to account for potential clinical confounding and population stratification.

Firth logistic regression was automatically applied when required using the “—glm hide-covar sex firth-fallback” option to improve estimation for low-frequency variants. Gene-based association testing was performed using MAGMA, which aggregates SNP-level association signals within genes while accounting for linkage disequilibrium to evaluate gene-level patterns of association. Gene boundaries were defined using the default MAGMA gene annotation. Genes with a nominal gene-based association *p* < 0.01 were selected for downstream functional enrichment and biological network analyses. Default MAGMA parameters were used throughout the analyses [14,24].

### 2.8. Functional Interpretation and Gene Interaction Network Analyses

Genes with a nominal gene-based association (*p* < 0.01) identified by MAGMA were subjected to downstream functional analyses to explore biological processes potentially associated with COVID-19 severity. Functional enrichment analysis was performed using Metascape (https://metascape.org), integrating Gene Ontology Biological Process, KEGG, Reactome, Hallmark gene sets, and Immunologic Signatures databases. Metascape default parameters were applied unless otherwise specified. Nominally enriched terms were identified using a minimum overlap of three genes, an enrichment factor > 1.5, and *p* < 0.01. Enriched terms were subsequently clustered according to functional similarity using the Metascape clustering algorithm to facilitate biological interpretation [15,17,18,25,26,27,28].

Gene interaction analysis was subsequently performed using GeneMANIA to investigate functional relationships among candidate genes based on co-expression, physical interactions, shared protein domains, pathway co-membership, predicted interactions, and genetic interactions (https://genemania.org); then the resulting interaction network was imported into Cytoscape (version 3.10.3) for visualization and network topology analysis [16,29,30]. Degree centrality was used to identify highly connected hub genes, whereas functional module assignment was performed through manual biological curation based on established gene functions and interaction patterns. Finally, the identified functional modules were integrated into a systems biology framework to facilitate mechanistic interpretation of host molecular responses associated with COVID-19 severity.

### 2.9. Statistical Analysis

Demographic and clinical characteristics were summarized using descriptive statistics. Continuous variables were compared using the independent-samples *t*-test or Mann–Whitney U test, whereas categorical variables were analyzed using the chi-square test or Fisher’s exact test, as appropriate. A two-sided *p* value < 0.05 was considered statistically significant.

Single-variant association analysis was performed using PLINK2 under an additive logistic regression model with adjustment for age, sex, ethnicity, and the first three principal components (PC1–PC3). Age and sex were obtained from the clinical records, whereas PC1–PC3 were derived from genome-wide genetic data and included to account for potential population stratification. After the main variant-level quality-control filters, 309,895 variants remained. Of these, 308,014 yielded valid regression *p*-values and were included in the single-variant association results and multiple-testing assessment. Gene-based association analysis was subsequently conducted using MAGMA, which aggregates SNP-level association signals within genes while accounting for linkage disequilibrium. Genes with a nominal gene-based *p* < 0.01 were selected solely for exploratory downstream functional enrichment and network analyses.

As sensitivity analyses, single-variant association analyses were repeated using two reduced covariate models: (i) age, sex, and ethnicity, with PC1–PC3 omitted; and (ii) age and sex only, with ethnicity and PC1–PC3 omitted. Effect estimates from these models were compared with those from the primary model.

Scenario-based post hoc power analysis was performed using QUANTO 1.2.4 to evaluate the ability of the study cohort (23 severe/critical and 24 mild/asymptomatic participants; *n* = 47) to detect single-variant associations under a range of minor allele frequencies (MAFs) and assumed odds ratios (ORs). Power was evaluated under a two-sided nominal significance level of α = 0.05 and under a Bonferroni-adjusted threshold of α = 1.62 × 10^−7^, calculated from 308,014 variants yielding valid regression *p*-values. The power assumptions and results are summarized in Supplementary Table S5.

### 2.10. Ethical Approval

The study protocol was approved by the Institutional Review Board for Biomedical Research of Hanoi Medical University under approval numbers 546/GCN-HDDDNCYSH-DHYHN. Written informed consent was obtained from all participants or their legally authorized representatives before enrollment. The study was conducted in accordance with the principles of the Declaration of Helsinki and applicable national regulations governing biomedical research involving human participants. Clinical data were obtained from participating healthcare institutions under formal authorization and anonymized before analysis. All genomic and clinical analyses were performed using coded datasets, and patient confidentiality was maintained throughout the study.

## 3. Results

### 3.1. Characteristics of the Study Population

A total of 48 Vietnamese patients with laboratory-confirmed COVID-19 were included in the clinical cohort. Based on clinical severity according to the Vietnamese Ministry of Health classification, participants were stratified into two groups comprising 24 mild/asymptomatic and 24 severe/critical cases. Baseline demographic and clinical characteristics of the 48 participants are summarized in Supplementary Table S1.

The two groups did not differ significantly in sex distribution (50.0% vs. 41.7% male, *p* = 0.56) or overall comorbidity rates (25.0% vs. 8.3%, *p* = 0.12). These clinical characteristics were assessed independently as descriptive and comparative analyses of the enrolled cohort. For the subsequent genetic association analyses, one participant was excluded during sample-level genetic quality control because the genetically inferred sex was discordant with the sex recorded in the medical record, leaving 47 participants (24 mild/asymptomatic and 23 severe/critical) for downstream genetic analyses. Age, sex, and ethnicity were then included as covariates in the genetic association models, together with PC1–PC3 derived from genome-wide genetic data.

**Figure 2:**
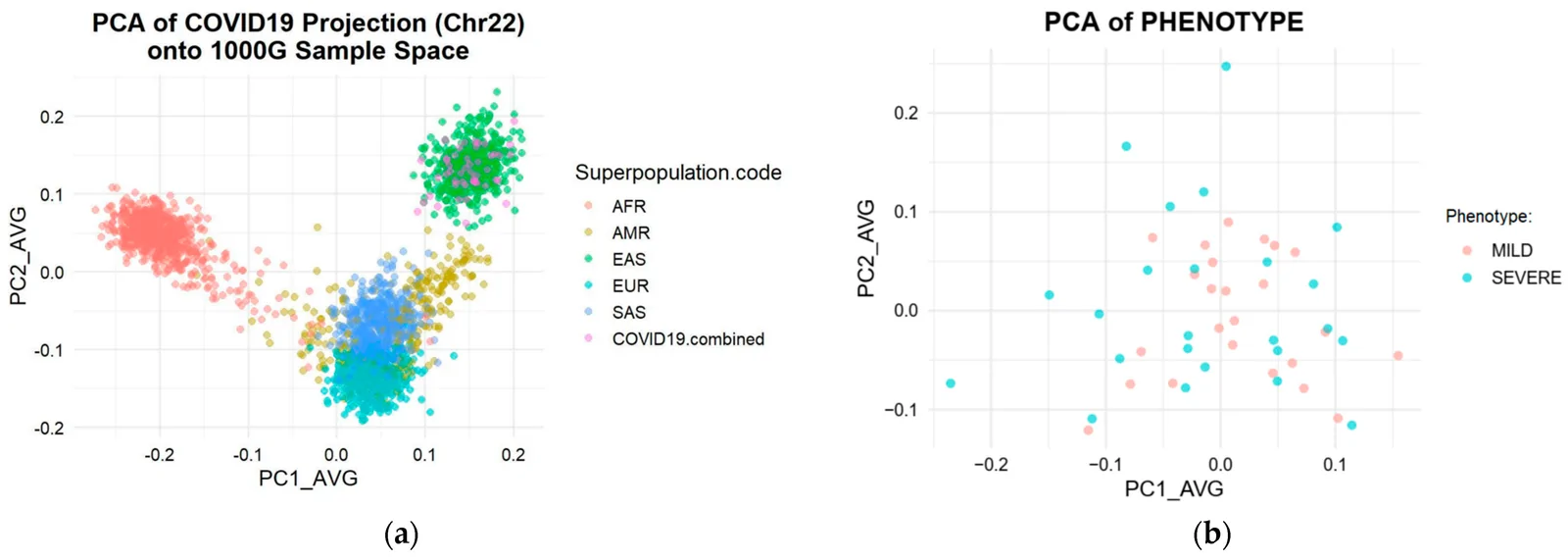
Principal component analysis (PCA) of the study population. PCA based on genome-wide genetic variants was performed to assess population genetic structure. (**a**) PCA plot combining the study cohort with reference populations from the 1000 Genomes Project. The Vietnamese study participants clustered closely within the East Asian (EAS) reference population, consistent with their expected genetic ancestry. (**b**) PCA plot of the study cohort stratified by COVID-19 severity. The substantial overlap between the mild/asymptomatic and severe/critical groups indicates no apparent population substructure associated with clinical severity. The first three principal components were subsequently included as covariates in the genetic association models.

Furthermore, principal component analysis (PCA) showed that the study participants clustered closely within the East Asian (EAS) reference population, with substantial overlap between the mild/asymptomatic and severe/critical groups and no apparent population substructure associated with disease severity (Figure 2).

### 3.2. Whole-Exome Sequencing Identifies Candidate Genes Associated with COVID-19 Severity

Whole-exome sequencing (WES) was successfully completed for all 48 enrolled patients. During sample-level genetic quality control, the sex recorded in the clinical medical record was compared with genetically inferred sex based on X-chromosome heterozygosity. One sample showed discordance between the reported and genetically inferred sex and was excluded from downstream genetic analyses. The remaining 47 samples (24 mild/asymptomatic and 23 severe/critical) passed the predefined genetic quality-control procedures and were retained for association analyses. The SNP-level association statistics used for MAGMA gene-based analysis were generated from additive logistic regression models adjusted for age, sex, and PC1–PC3.

No individual variant reached the Bonferroni-adjusted significance threshold of α = 1.62 × 10^−7^. Gene-based analysis was subsequently performed using MAGMA to aggregate SNP-level association signals within genes and evaluate gene-level patterns of association. Population genetic structure was further evaluated by PCA. The study participants clustered closely within the East Asian (EAS) reference population, with substantial overlap between the mild/asymptomatic and severe/critical groups (Figure 2). These findings indicate no apparent population substructure associated with clinical severity. PC1–PC3 were nevertheless retained as covariates in the genetic association models to account conservatively for potential population stratification.

**Figure 3:**
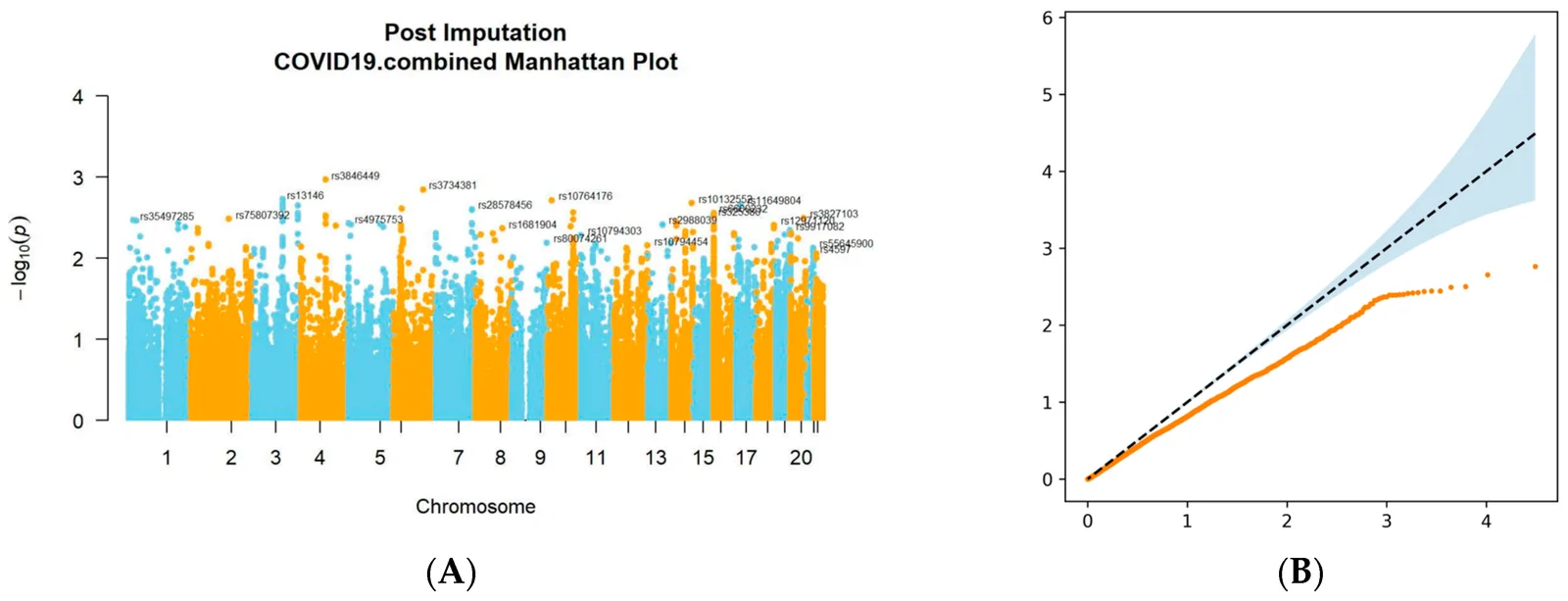
Gene-based association analysis using MAGMA. (**A**) Manhattan plot showing gene-level association signals across the genome. The horizontal dashed line indicates the predefined nominal significance threshold (*p* = 0.01) used to select candidate genes for downstream analyses. (**B**) Quantile–quantile (Q–Q) plot comparing observed and expected gene-based association statistics. The overall distribution showed no marked deviation from the null expectation across most of the distribution, suggesting minimal systematic inflation. The observed—log10 (*p*-value) from the MAGMA analysis (blue area) are plotted against the expected null distribution. The dashed gray line represents the theoretical diagonal under the null hypothesis, and the shaded gray area indicates the 95% confidence envelope. The gene-level genomic inflation factor (λ (gene-level) = 0.944) did not indicate substantial systematic inflation of the gene-based association statistics.

The distribution of gene-based association statistics is presented in the Manhattan and quantile–quantile (Q–Q) plots (Figure 3). The Manhattan plot demonstrated that association signals were distributed across multiple genomic regions rather than concentrated within a single susceptibility locus, whereas the Q–Q plot showed close agreement between observed and expected statistics throughout most of the distribution, with deviation confined to the upper tail, consistent with a limited subset of potentially associated genes.

After the main variant-level quality-control filters, 309,895 variants remained available for association testing. Of these, 308,014 variants yielded valid regression *p*-values from the PLINK2 analysis and were included in the single-variant multiple-testing assessment. No individual variant reached the Bonferroni-adjusted significance threshold of α = 1.62 × 10^−7^.

Gene-based analysis was subsequently performed using MAGMA. A total of 15,465 genes were evaluated, and 44 genes met the nominal gene-based prioritization threshold of *p* < 0.01 (Supplementary Table S3). None of these genes remained significant after Benjamini–Hochberg correction; the adjusted *p*-values were approximately 0.9994. Accordingly, these 44 genes were retained solely for exploratory downstream functional and network analyses.

Sensitivity analyses were performed to assess the influence of covariate specification on single-variant effect estimates. Comparison of the primary model with the age-, sex-, and ethnicity-adjusted model showed high concordance in effect estimates (Pearson’s r = 0.891; Spearman’s ρ = 0.885; median absolute difference in log(OR) = 0.210). Comparison with the age- and sex-adjusted model showed moderate-to-high concordance (Pearson’s r = 0.772; Spearman’s ρ = 0.781; median absolute difference in log(OR) = 0.296). These findings suggest that effect estimates were broadly concordant across covariate specifications, although nominal statistical significance was less stable in the reduced age- and sex-only model.

Using a predefined threshold of gene-based *p* < 0.01, 44 candidate genes were identified and selected for downstream analyses (Supplementary Table S3). These genes were distributed across multiple chromosomes rather than clustered within a single genomic locus, a pattern that is consistent with a potentially polygenic contribution to COVID-19 severity. The candidate genes were subsequently subjected to functional enrichment and gene interaction network analyses, including GO, KEGG, Reactome, MSigDB Hallmark, Immunologic Signatures, GeneMANIA, and Cytoscape, to characterize their shared biological functions, interaction patterns, and systems-level organization.

### 3.3. Functional Enrichment Analysis Reveals Immune- and Inflammation-Related Biological Processes Associated with COVID-19 Severity

**Figure 4:**
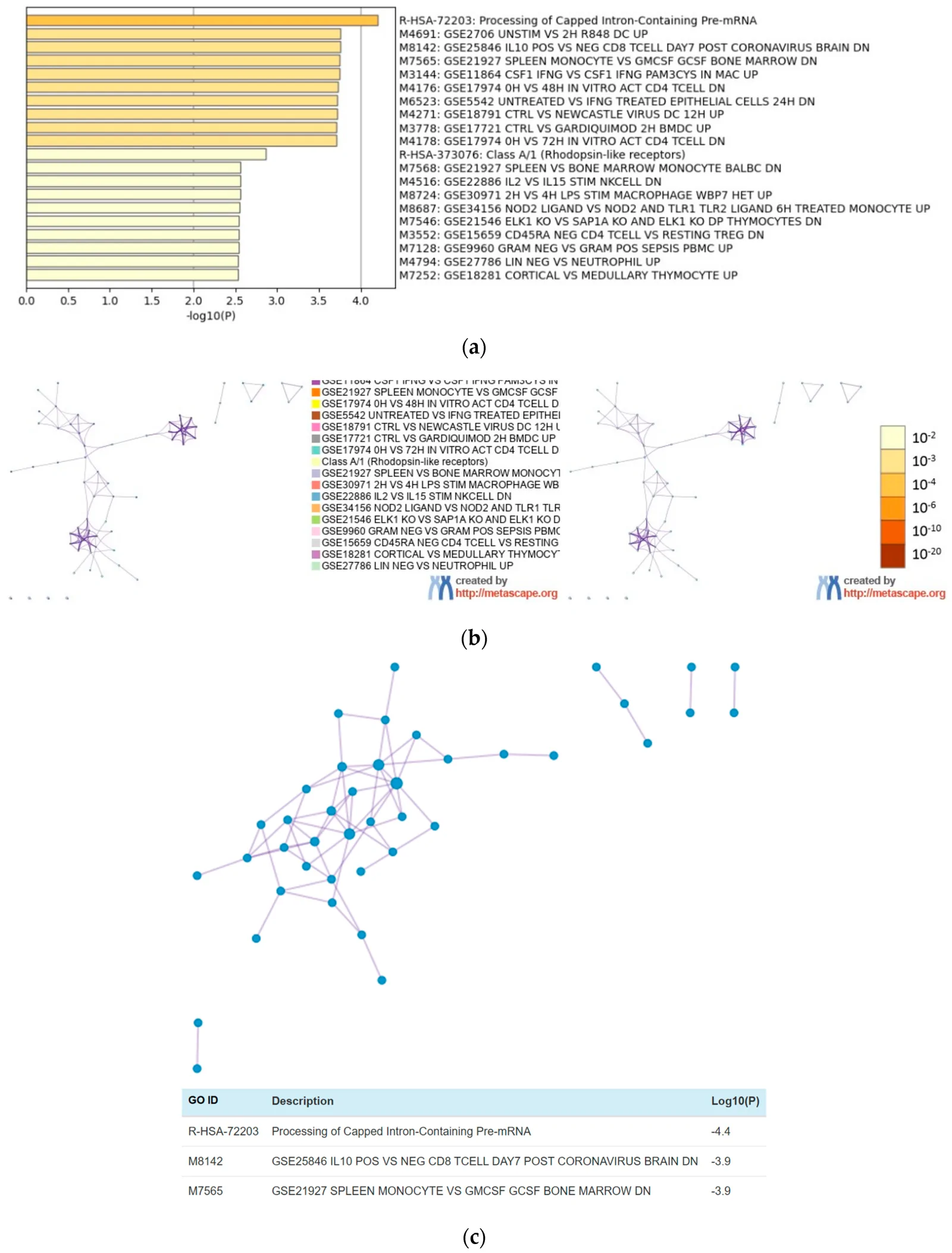
Functional enrichment analysis of candidate genes associated with COVID-19 severity. Functional enrichment analysis of the 44 candidate genes identified by MAGMA using Metascape: (**a**) Top enriched biological terms identified from integrated GO, KEGG, Reactome, MSigDB Hallmark, and Immunologic Signatures databases. Circle size represents the number of genes associated with each enriched term, whereas color intensity corresponds to statistical significance. (**b**) Enrichment similarity network illustrating relationships among significantly enriched biological terms. Each node represents an enriched term, with edges indicating similarity based on shared gene membership. Clusters of related terms are shown in different colors. (**c**) Representative MCODE protein–protein interaction (PPI) module together with its top enriched biological functions, highlighting densely connected subnetworks identified from the candidate genes.

To investigate the biological significance of the 44 candidate genes identified by gene-based association analysis, functional enrichment analysis was performed using Metascape. Significant enrichment was observed across multiple complementary functional annotation databases, including GO Biological Process, KEGG, Reactome, MSigDB Hallmark, and Immunologic Signatures (Figure 4). GO enrichment analysis demonstrated that the candidate genes were predominantly involved in RNA processing and mRNA metabolic processes, including RNA splicing, mRNA maturation, and regulation of RNA stability, indicating that post-transcriptional gene regulation may contribute substantially to host responses during SARS-CoV-2 infection. Reactome pathway analysis further highlighted enrichment of pathways associated with mRNA processing, mitochondrial translation, respiratory electron transport, and cellular energy metabolism, whereas KEGG analysis identified pathways related to mitochondrial function and cellular metabolic regulation.

Consistent with these findings, Hallmark gene sets demonstrated significant enrichment of pathways associated with cellular stress responses and metabolic adaptation. Immunologic Signatures additionally suggested that several candidate genes participate in immune-related transcriptional programs involved in host antiviral responses.

Collectively, the integrated Metascape analysis demonstrated that the candidate genes converged on biological processes involving RNA metabolism, mitochondrial function, cellular metabolism, and immune regulation. The enrichment similarity network further revealed that these pathways formed closely interconnected functional clusters rather than independent biological processes, whereas MCODE analysis identified highly connected protein interaction modules supporting coordinated cellular responses (Figure 4).

To assess the sensitivity of the functional findings to the gene-selection threshold, enrichment analysis was repeated using a broader set of 368 genes meeting nominal MAGMA *p* < 0.05. The major RNA-processing theme identified in the primary analysis was preserved, with the Reactome pathway ‘Processing of Capped Intron-Containing Pre-mRNA’ remaining among the top enriched pathways and increasing from 5 to 15 contributing genes. Immune-related signatures and GPCR-associated signaling were also retained across the two thresholds (Supplementary Table S6). These results indicate that several major exploratory functional themes were consistent across alternative gene-selection thresholds.

### 3.4. Functional Clustering Identifies Coordinated Biological Modules Associated with COVID-19 Severity

**Figure 5:**
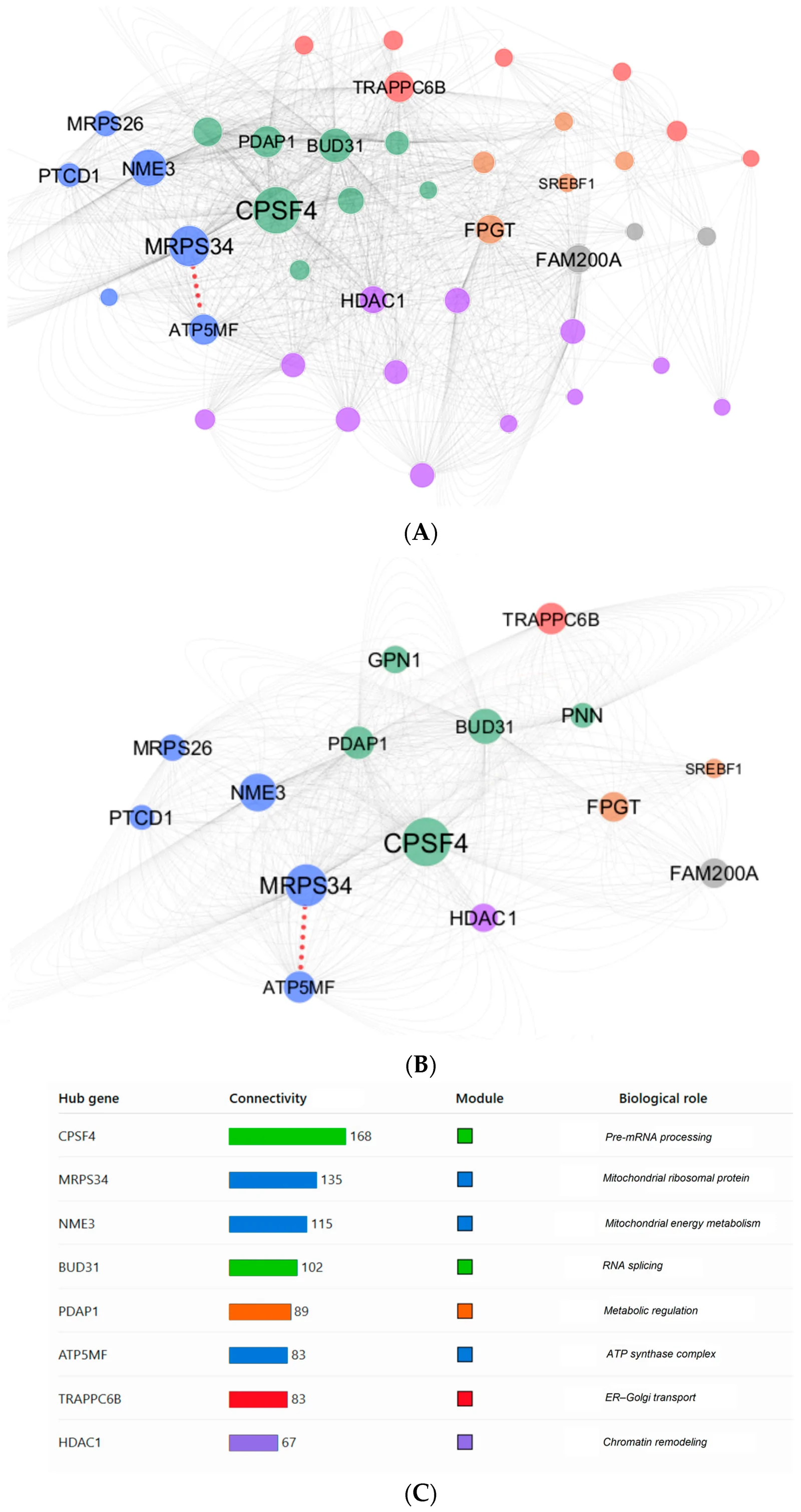
Gene interaction network of candidate genes associated with COVID-19 severity. (**A**) GeneMANIA interaction network of the 44 candidate genes. Node colors indicate functional modules, including RNA processing (blue), mitochondrial bioenergetics (orange), metabolic reprogramming (green), transcriptional and epigenetic regulation (purple), membrane trafficking and cell communication (red), and cellular homeostasis (gray). (**B**) Subnetwork highlighting the major hub genes identified by network topology analysis. (**C**) Functional module organization and hub-gene architecture visualized in Cytoscape. Node size is proportional to degree centrality.

To facilitate biological interpretation, the enriched pathways and candidate genes were further organized into six major functional modules according to their predominant biological functions (Figure 5). These modules comprised RNA processing, mitochondrial bioenergetics, metabolic reprogramming, transcriptional and epigenetic regulation, membrane trafficking and cell communication, and cellular homeostasis. The RNA processing module contained several highly connected genes involved in pre-mRNA splicing, RNA maturation, and post-transcriptional regulation, including BUD31, CPSF4, SNRPD3, PNN, and YLPM1. The mitochondrial bioenergetics module included genes associated with mitochondrial protein translation and oxidative phosphorylation, such as ATP5MF, MRPS34, MRPS26, PTCD1, NME3, and MGARP. Genes involved in metabolic reprogramming, including PDAP1, FPGT, UMPS, and SREBF1, suggested altered cellular metabolic adaptation during severe disease.

A fourth module consisted of genes associated with transcriptional and epigenetic regulation, including HDAC1, PHF14, PKNOX1, and multiple zinc-finger proteins, supporting widespread transcriptional remodeling. Genes participating in membrane trafficking and cell communication, including TRAPPC6B, VPS29, GET4, SUN1, and NUP160, indicated potential disruption of intracellular transport and nucleocytoplasmic trafficking. Finally, a cellular homeostasis module comprised genes involved in diverse regulatory functions that collectively contribute to maintenance of cellular integrity.

Although each module represents a distinct biological process, substantial functional overlap was observed among them, suggesting coordinated host responses to SARS-CoV-2 infection rather than independent molecular events.

### 3.5. Gene Interaction Network Analysis Identifies Highly Connected Hub Genes Underlying the Candidate Gene Network

Gene interaction network analysis using GeneMANIA demonstrated extensive functional connectivity among the 44 candidate genes through multiple evidence types, including co-expression, physical interactions, predicted interactions, co-localization, shared protein domains, shared pathways, and genetic interactions (Figure 5A). The network was highly interconnected and lacked a single dominant regulatory node, supporting the polygenic nature of host genetic susceptibility to severe COVID-19.

Network topology analysis performed in Cytoscape identified several highly connected hub genes with prominent positions within the interaction network (Figure 5B,C). CPSF4, BUD31, and SNRPD3 represented the highly connected genes in the inferred interaction network of the RNA processing module, whereas ATP5MF, MRPS34, PTCD1, and NME3 formed the core of the mitochondrial bioenergetics module. Additional hub genes, including PDAP1, HDAC1, FPGT, and TRAPPC6B, connected multiple functional modules related to cellular metabolism, transcriptional regulation, intracellular trafficking, and maintenance of cellular homeostasis.

Visualization of the network according to manually curated functional modules further demonstrated that hub genes were distributed across all six biological modules rather than confined to a single pathway (Figure 5C). These observations indicate that the candidate genes constitute an integrated molecular interaction network involving multiple coordinated biological processes associated with COVID-19 severity.

### 3.6. Integrated Overview of the Biological Findings

**Figure 6:**
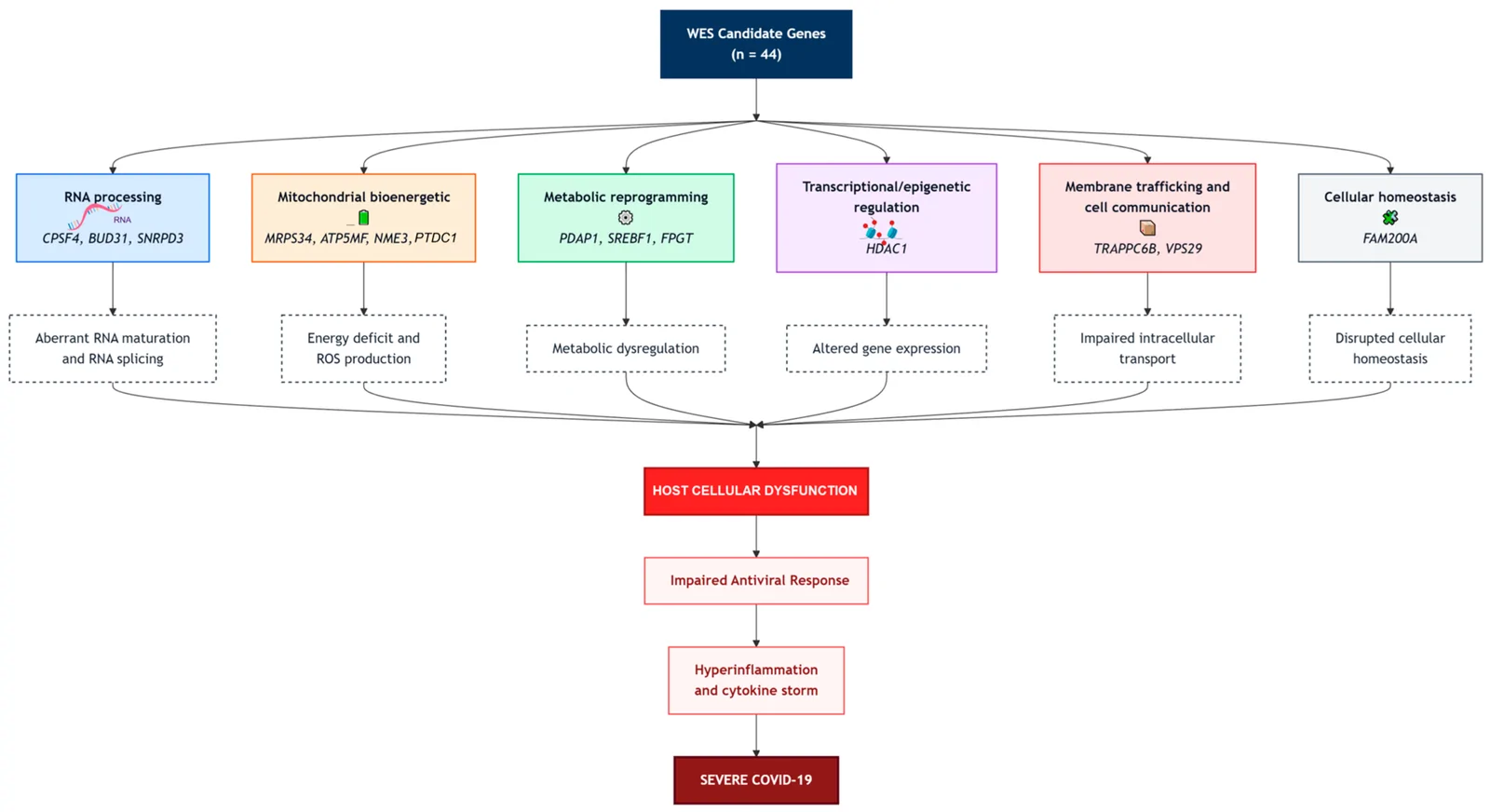
Hypothesized biological framework underlying host genetic susceptibility to severe COVID-19. Gene-based association, functional enrichment, and network analyses were integrated to construct a systems biology model illustrating how six interconnected functional modules may collectively be relevant to host cellular responses associated with severe COVID-19. These modules converge on dysregulated RNA processing, impaired mitochondrial bioenergetics, metabolic reprogramming, transcriptional and epigenetic remodeling, altered membrane trafficking and cell communication, and disruption of cellular homeostasis, ultimately potentially promoting immune dysregulation and increased disease severity. Conceptual, hypothesis-generating model integrating functionally related candidate genes and pathways identified in the present exploratory analysis. The model does not imply experimentally demonstrated causality or directionality.

The results of gene-based association, functional enrichment, and gene interaction network analyses were integrated into a hypothesized biological framework summarizing the potential molecular processes associated with COVID-19 severity (Figure 6).

The model illustrates how the six functional modules identified in the present study—including RNA processing, mitochondrial bioenergetics, metabolic reprogramming, transcriptional and epigenetic regulation, membrane trafficking and cell communication, and cellular homeostasis—are interconnected rather than functioning independently. Hub genes identified from the interaction network may provide potential molecular links among these biological processes, which are likely to be functionally interconnected rather than acting independently. Collectively, these analyses provide a conceptual, hypothesis-generating framework rather than definitive evidence of causal mechanisms.

## 4. Discussion

### 4.1. Principal Findings

The genetic basis underlying the marked interindividual variability in COVID-19 severity remains incompletely understood despite major advances from genome-wide association studies and international collaborative efforts [7,9,10,31]. Current evidence indicates that severe COVID-19 is a complex polygenic disorder in which susceptibility arises from coordinated perturbations across multiple biological systems rather than defects in individual immune-related genes [7,31,32].

In the present pilot study, whole-exome sequencing combined with gene-based association analysis and integrative systems biology identified 44 genes prioritized for exploratory downstream analyses based on nominal MAGMA *p* < 0.01 in unvaccinated Vietnamese patients infected with the SARS-CoV-2 Delta variant. Rather than identifying a single major susceptibility locus, the Manhattan plot demonstrated that association signals were distributed across multiple genomic regions, consistent with a potentially polygenic contribution to COVID-19 severity. Furthermore, the absence of substantial inflation in the gene-based association statistics (Figure 3B) provides no evidence of substantial systematic inflation of the gene-based association statistics; PCA similarly showed no apparent population substructure associated with clinical severity. Functional enrichment and gene interaction network analyses consistently demonstrated that these candidate genes converged on multiple interconnected biological processes involving RNA processing, mitochondrial bioenergetics, metabolic reprogramming, transcriptional and epigenetic regulation, membrane trafficking and cell communication, and cellular homeostasis. This systems-level organization is consistent with the principles of network medicine, whereby disease susceptibility results from perturbations across interacting molecular networks rather than isolated genes [32,33,34].

Among these biological processes, RNA processing and mRNA maturation emerged as the predominant functional module. This observation is biologically plausible because SARS-CoV-2 extensively exploits host RNA-processing machinery throughout its replication cycle while simultaneously reshaping host RNA splicing and post-transcriptional regulation to facilitate viral replication and evade antiviral immunity [35,36]. In parallel, our analyses also highlighted mitochondrial dysfunction, metabolic reprogramming, and transcriptional regulation, consistent with increasing evidence implicating these processes in immune dysregulation and COVID-19 pathogenesis [37,38,39].

Given the exploratory nature of this pilot WES study and the relatively limited genetic analysis set (23 severe and 24 mild cases after genetic quality control), the present analysis was not powered to detect variants with modest effect sizes at exome-wide significance. Consequently, the identified candidate genes should be considered hypothesis-generating and require validation in larger independent cohorts. Compared with the previous Vietnamese WES study, which primarily reported susceptibility genes associated with COVID-19 severity [39], our study extends these observations by integrating gene-based association analysis with functional enrichment, interaction network analysis, and systems biology modeling to provide a more comprehensive biological interpretation of host genetic susceptibility. Among the six functional modules identified, the RNA-processing module exhibited the highest degree of functional connectivity and network centrality, suggesting that host RNA metabolism may represent a biologically plausible pathway for further investigation in relation to severe COVID-19.

### 4.2. RNA Processing and mRNA Maturation Emerge as the Predominant Biological Module Associated with Severe COVID-19

One of the principal findings of this study was the identification of a densely interconnected RNA-processing module, comprising BUD31, CPSF4, SNRPD3, PNN, NUP160, GPN1, YLPM1, VPS29, GET4, and SUN1. Network topology analysis further identified CPSF4, BUD31, and SNRPD3 as the major hub genes within this module, suggesting that RNA-processing pathways may represent plausible candidates for further investigation in relation to host susceptibility to severe COVID-19.

This observation is biologically plausible because SARS-CoV-2 relies extensively on host RNA metabolism throughout its replication cycle. Viral transcription, RNA processing, protein synthesis, and modulation of innate antiviral responses all depend on coordinated interactions with host transcriptional and post-transcriptional regulatory machinery. Consequently, disruption of RNA splicing, mRNA maturation, nuclear export, or transcript stability may influence both viral replication efficiency and the magnitude of host immune responses [35,39,40,41,42,43].

The three hub genes identified in this study perform complementary functions within the RNA-processing pathway. BUD31 is an essential spliceosomal component required for pre-mRNA splicing, SNRPD3 participates in spliceosome assembly as a core small nuclear ribonucleoprotein (snRNP), whereas CPSF4 is a key component of the cleavage and polyadenylation specificity factor complex responsible for mRNA 3′-end processing. Although these genes have not consistently emerged as major COVID-19 susceptibility loci in previous genetic studies, their central positions within the interaction network suggest that coordinated perturbation of RNA-processing pathways, rather than dysfunction of individual genes, may exert widespread downstream effects on antiviral immunity and cellular homeostasis [35,44,45]. Notably, our network analysis identified CPSF4 (cleavage and polyadenylation specificity factor 4) as the most prominent hub within the RNA-processing machinery. During SARS-CoV-2 infection, viral proteins (such as NSP1) are known to globally disrupt host mRNA processing and nuclear export to shut down the host’s antiviral defense. Genetic variation affecting CPSF4 could theoretically influence mRNA 3′-end processing and transcript stability; however, the present study did not directly assess CPSF4 function or interferon-stimulated gene expression. This possibility therefore requires experimental validation.

Recent transcriptomic and proteomic studies have demonstrated that SARS-CoV-2 extensively remodels host RNA metabolism by altering alternative splicing, mRNA stability, RNA modification, and host gene expression to suppress antiviral defenses while facilitating viral replication [35,41,42,43,46]. In addition, accumulating evidence indicates that RNA-processing machinery also regulates immune-cell differentiation, interferon signaling, inflammatory responses, and stress adaptation [39,47,48].

Our findings extend these observations by suggesting that inherited variation affecting host RNA-processing pathways may contribute to inter-individual differences in COVID-19 severity. Collectively, these results identify RNA processing and mRNA maturation as the predominant functional theme in the exploratory enrichment analysis in this cohort and provide a biological rationale for future functional studies investigating the contribution of post-transcriptional regulation to host antiviral immunity.

### 4.3. Mitochondrial Bioenergetics and Energy Metabolism May Contribute to Disease Progression

Another major functional module identified in this study comprised genes involved in mitochondrial bioenergetics and energy metabolism, including ATP5MF, PTCD1, MRPS26, MRPS34, MGARP, and NME3. Network topology analysis further identified ATP5MF, MRPS34, PTCD1, and NME3 as central components of this module, suggesting that inherited variation affecting mitochondrial homeostasis may contribute to inter-individual differences in host responses to SARS-CoV-2 infection.

Mitochondria are central regulators of cellular bioenergetics and play indispensable roles in antiviral immunity through ATP production, reactive oxygen species (ROS) generation, mitochondrial antiviral signaling (MAVS), apoptosis, inflammasome activation, and type I interferon responses [37,49,50]. Consequently, disruption of mitochondrial integrity or oxidative phosphorylation may impair the ability of immune cells to mount effective antiviral responses while simultaneously promoting excessive inflammatory activation. Increasing evidence indicates that mitochondrial dysfunction represents a characteristic feature of severe COVID-19 and contributes to immune dysregulation, cytokine production, and tissue injury [37,38,51]. The genes identified in the present study further support this biological interpretation. ATP5MF encodes a subunit of mitochondrial ATP synthase that is essential for oxidative phosphorylation and ATP production, whereas PTCD1 regulates mitochondrial RNA metabolism and respiratory chain function. MRPS26 and MRPS34 encode mitochondrial ribosomal proteins required for intramitochondrial protein translation, while NME3 and MGARP contribute to mitochondrial organization and maintenance. Although none of these genes has consistently emerged as a major COVID-19 susceptibility locus, their coordinated enrichment within a single functional module suggests that inherited variation affecting mitochondrial bioenergetics may exert cumulative biological effects on host responses to SARS-CoV-2 infection rather than acting through individual genes alone. This interpretation is consistent with the underlying rationale of gene-based association analysis and systems biology, in which multiple variants with individually modest effects collectively perturb functionally related biological pathways. The identification of mitochondrial ribosomal proteins, specifically MRPS34 and MRPS26, further strengthens this biological link. The enrichment of mitochondrial ribosomal proteins provides a biologically plausible connection to mitochondrial homeostasis and antiviral signaling. However, the present WES data do not directly demonstrate mitochondrial depolarization, altered MAVS activity, or impaired type I interferon signaling in the study participants. These potential mechanistic links therefore remain speculative and should be regarded as hypotheses for future functional investigation rather than as mechanisms demonstrated by the present study.

Recent immunometabolic studies further demonstrate that metabolic flexibility is essential for appropriate activation of lymphocytes and monocytes during viral infection, requiring dynamic coordination between oxidative phosphorylation and glycolytic metabolism [38,52]. Mitochondria therefore occupy a central position linking cellular energy production with innate immune signaling, and disruption of mitochondrial homeostasis has increasingly been recognized as a major driver of immunometabolic dysfunction during severe COVID-19 [53]. Our findings therefore support the hypothesis that inherited variation affecting mitochondrial function may influence immunometabolic capacity and consequently modify the clinical severity of COVID-19. Future functional studies are warranted to determine whether these genes directly regulate antiviral immunity or primarily influence disease progression through maintenance of mitochondrial homeostasis.

### 4.4. Metabolic Reprogramming and Transcriptional Regulation in Severe COVID-19

Another functional module identified in our analyses comprised genes associated with metabolic adaptation and transcriptional regulation, including SREBF1, HDAC1, MC3R, FGFBP3, PHF14, PKNOX1, and several zinc-finger proteins. Although these genes perform diverse biological functions, they converge on pathways governing metabolic homeostasis, transcriptional control, and cellular adaptation, suggesting that coordinated immunometabolic remodeling contributes to COVID-19 severity.

SREBF1 is a master transcriptional regulator of cholesterol and fatty acid biosynthesis. Increasing evidence indicates that SARS-CoV-2 extensively remodels host lipid metabolism to support viral replication, membrane biogenesis, and immune evasion, whereas dysregulated cholesterol metabolism also contributes to inflammatory signaling and immune dysfunction [54,55,56]. These observations support the possibility that inherited variation affecting lipid metabolic pathways may influence susceptibility to severe disease.

HDAC1 represents one of the major epigenetic regulators identified in the present study. Histone deacetylases regulate chromatin remodeling, interferon signaling, inflammatory gene expression, and immune-cell differentiation, and increasing evidence suggests that SARS-CoV-2 infection induces extensive epigenetic remodeling of host immune cells [57,58,59,60]. PHF14 and PKNOX1 further support this regulatory module through their roles in chromatin-associated transcriptional regulation and cellular differentiation, whereas the zinc-finger proteins identified in our dataset likely contribute to fine-tuning gene expression rather than serving as primary regulators of antiviral immunity [61].

Although MC3R and FGFBP3 have not been extensively investigated in COVID-19, both genes participate in systemic metabolic regulation and inflammatory homeostasis. Their identification in the present study raises the possibility that inherited variation affecting metabolic adaptation may influence disease severity indirectly through regulation of host energy balance and immune function rather than direct antiviral activity.

Importantly, our findings are consistent with the emerging concept of immunometabolic reprogramming, in which immune cells undergo coordinated metabolic remodeling during SARS-CoV-2 infection, including enhanced glycolysis, altered lipid metabolism, mitochondrial dysfunction, anabolic reprogramming, and epigenetic regulation required to sustain antiviral responses [38,50,52,62,63]. Our results suggest that this functional module acts in concert with RNA-processing and mitochondrial pathways to regulate the balance between protective antiviral immunity and pathological inflammation.

### 4.5. Transcriptional Regulation and Nuclear Organization as Upstream Candidate Genes of Host Responses

A fourth functional module identified in our integrative analyses comprised genes involved in transcriptional regulation, chromatin organization, and nuclear architecture, including PHF14, PKNOX1, CEP85L, CCDC121, EME2, ZNF655, ZFP28, and ZNF470. Although these genes have not been widely recognized as COVID-19 susceptibility genes, they share regulatory functions in gene expression and chromatin dynamics, suggesting that inherited variation affecting transcriptional control may contribute to inter-individual differences in host responses to SARS-CoV-2 infection.

Among these genes, PHF14 and PKNOX1 occupied relatively central positions within the interaction network. PHF14 is a chromatin-associated regulator involved in transcriptional control, cellular differentiation, and responses to cellular stress and hypoxia, processes relevant to severe viral pneumonia [64]. PKNOX1 encodes a TALE-class homeobox transcription factor that coordinates multiple developmental and immune-related transcriptional programs. Although its direct involvement in COVID-19 has not been established, variation affecting transcriptional regulators such as PKNOX1 could broadly influence host-response programs during viral infection. These observations are consistent with recent systems biology studies showing that SARS-CoV-2 extensively remodels host transcriptional regulatory networks and chromatin organization, affecting inflammatory signaling, interferon responses, cell-cycle regulation, and stress-response pathways [65,66,67]. In addition, the enrichment of several zinc-finger proteins (ZNF655, ZFP28, and ZNF470) further supports the importance of transcriptional regulation, as members of this protein family have previously been implicated in antiviral immunity [68,69].

Collectively, this module suggests that inherited variation affecting chromatin organization and transcriptional regulation may influence multiple downstream biological pathways by shaping the capacity of host cells to coordinate appropriate transcriptional responses following SARS-CoV-2 infection. Within the integrated systems biology framework proposed in the present study, these genes are therefore more likely to function as upstream regulators of host cellular responses than as independent candidate genes of disease severity.

### 4.6. Cell Communication, Membrane Trafficking, and Cellular Stress Adaptation

The final functional module identified in our network analysis comprised genes involved in cell communication, membrane trafficking, vesicular transport, and cellular stress adaptation, including CCRL2, CALHM2, TACR1, TRAPPC6B, SERPINB11, OR2F1, LHX9, CTXN3, FPGT, FAM200A, PROX2, and UMPS. Although functionally heterogeneous, these genes participate in biological processes that coordinate intercellular communication, intracellular trafficking, and cellular adaptation to physiological stress.

Among these genes, CCRL2 has the strongest immunological relevance. As an atypical chemokine receptor regulating leukocyte migration and inflammatory cell recruitment, CCRL2 has been implicated in neutrophil and macrophage trafficking, endothelial activation, and local inflammatory responses. Given that dysregulated chemokine signaling is a hallmark of severe COVID-19, inherited variation affecting CCRL2 may influence pulmonary inflammation and disease severity [70].

Another notable gene, TRAPPC6B, encodes a component of the transport protein particle (TRAPP) complex involved in intracellular vesicle trafficking. Although not directly associated with COVID-19 susceptibility, membrane trafficking is essential for the SARS-CoV-2 life cycle, including replication organelle formation, virion assembly, and viral egress. Consequently, inherited variation affecting vesicular transport may modify viral replication efficiency or host cellular responses [71,72,73].

Additional genes, including CALHM2, TACR1, and UMPS, are involved in calcium homeostasis, neuroimmune signaling, and nucleotide metabolism. Although direct evidence linking these genes to COVID-19 remains limited, they participate in cellular processes associated with adaptation to metabolic stress and sustained viral infection. Recent systems biology studies likewise demonstrate that SARS-CoV-2 extensively remodels host membrane organization, vesicular transport, and intracellular signaling networks, supporting these pathways as potential modifiers of disease severity [69,71,74,75].

Taken together, this heterogeneous functional module further emphasizes that host susceptibility to severe COVID-19 extends beyond classical antiviral or inflammatory pathways. Rather, efficient host defense appears to depend on coordinated interactions among multiple cellular systems—including membrane trafficking, intracellular communication, metabolic adaptation, and stress-response pathways—which collectively maintain cellular homeostasis during viral infection.

### 4.7. An Integrated Systems Biology Model of Host Genetic Susceptibility to Severe COVID-19

Rather than implicating a single biological pathway, our findings suggest that severe COVID-19 results from coordinated perturbations across multiple interconnected cellular systems. Integration of gene-based association analysis, functional enrichment, and interaction network reconstruction identified six major functional modules that together provide a systems-level framework for understanding host genetic susceptibility in Vietnamese patients infected with the SARS-CoV-2 Delta variant.

Within this framework, RNA processing and mRNA maturation represent the primary upstream regulatory module, potentially influencing antiviral transcriptional responses before activation of classical immune pathways. Mitochondrial bioenergetics and immunometabolism constitute a second regulatory layer that supports immune-cell activation through coordinated ATP production, mitochondrial gene expression, and metabolic adaptation. Inherited variation affecting these processes may impair antiviral immunity while promoting oxidative stress and inflammatory signaling [47,49,50].

These upstream mechanisms converge with metabolic reprogramming and epigenetic regulation, represented by genes such as SREBF1 and HDAC1, which coordinate lipid metabolism, chromatin remodeling, and transcriptional plasticity. SARS-CoV-2 is known to extensively remodel host metabolic and epigenetic pathways to facilitate viral replication and modulate inflammatory responses [38,55,57,58,59,60,62]. Downstream, transcriptional regulation, nuclear organization, membrane trafficking, and cellular stress adaptation collectively shape the intracellular environment that determines the magnitude and balance of antiviral responses.

To properly contextualize the 44 candidate genes identified in our gene-based analysis—none of which retained genome-wide significance after stringent false discovery rate (FDR) correction—their biological relevance should be evaluated within the framework of the omnigenic model of complex traits. Under this paradigm, host susceptibility to severe infection is driven not only by a few major core genes, but also by the cumulative burden of genetic variants distributed across interconnected functional networks. Although individual variant signals may remain sub-threshold due to cohort size limitations, their collective convergence on specific biological modules may provide a biologically plausible framework for interpreting sub-threshold signals, although this interpretation remains exploratory and requires independent validation. Importantly, our findings regarding RNA processing and mitochondrial bioenergetics (e.g., BUD31, CPSF4, ATP5MF, MRPS34) are broadly consistent with established functional and transcriptomic evidence of SARS-CoV-2 pathogenesis [35,76,77]. Experimental studies have demonstrated that viral non-structural proteins (e.g., NSP1) and accessory factors (e.g., ORF9b) directly target host RNA splicing machinery and disrupt mitochondrial TOM70/MAVS to evade innate immunity [78]. Thus, while our WES findings represent an exploratory hypothesis, they provide complementary host genomic observations that are biologically consistent with mitochondrial dysfunction and dysregulated RNA metabolism in severe COVID-19 [32,79].

Importantly, this systems-level framework must be interpreted strictly as a theoretical and hypothesis-generating model rather than an experimentally validated causal mechanism [32,33,34]. Because bioinformatic tools such as GeneMANIA and Metascape inherently construct interaction networks based on input gene lists regardless of effect sizes, functional validation studies are required to confirm whether these candidate genes directly drive host cellular dysfunction or represent secondary cellular responses. Future studies integrating transcriptomics, proteomics, metabolomics, and functional assays will be required to validate these biological pathways and clarify their roles in COVID-19 pathogenesis. Nevertheless, this framework provides a biologically coherent foundation for future mechanistic investigations into host genetic susceptibility to COVID-19. Finally, a critical bioinformatic caveat must be recognized regarding network construction algorithms. Software tools such as GeneMANIA and Metascape inherently rely on pre-existing knowledgebases and will algorithmically generate interconnected networks and enriched biological pathways whenever provided with a gene list, regardless of the statistical strength or causality of the input signals. Consequently, the dense topology and high degree centrality observed in our RNA processing and mitochondrial bioenergetics modules may partially reflect well-annotated literature biases within public databases rather than an exclusive host pathogenesis mechanism unique to this clinical cohort.

### 4.8. Comparison with Previous Genetic Studies of COVID-19

Our findings are consistent with growing evidence indicating that host susceptibility to severe COVID-19 is governed by complex potentially relevant pathways rather than single high-impact genetic variants. Large genome-wide association studies conducted by the COVID-19 Host Genetics Initiative have identified susceptibility loci associated with antiviral immunity, pulmonary injury, inflammatory signaling, and epithelial barrier function [7,9,31]. However, because GWASs primarily detect common variants, they provide limited insight into the cumulative effects of rare or low-frequency coding variants that can be identified by whole-exome sequencing.

Whole-exome sequencing therefore provides a complementary strategy for investigating protein-coding variation with potentially greater functional consequences. Nevertheless, published WES studies of COVID-19 have generally been constrained by relatively small sample sizes and substantial heterogeneity in ancestry, vaccination status, circulating viral variants, and clinical characteristics. Consequently, concordance at the level of individual susceptibility genes has been limited across studies, whereas convergence at the level of biological pathways has been considerably more consistent, reinforcing the concept that functional networks and disease modules may provide more informative units of biological interpretation than individual susceptibility genes [32,33,34]. This systems-level perspective is increasingly recognized as a fundamental principle of network medicine and complex disease biology [80,81].

Our findings also extend the previous Vietnamese WES study by Nhung et al. [13]. Whereas the earlier study primarily identified candidate susceptibility genes, we integrated gene-based association analysis with functional enrichment, interaction network reconstruction, and module-based systems biology to identify convergent biological processes involving RNA processing, mitochondrial bioenergetics, metabolic reprogramming, transcriptional regulation, and cellular homeostasis. This integrative strategy provides a broader biological context for interpreting candidate genes and generates testable mechanistic hypotheses for future functional studies. A notable strength of our cohort is its biological homogeneity. All participants were Vietnamese, infected with the SARS-CoV-2 Delta variant, unvaccinated before infection, and classified using standardized national clinical criteria. These characteristics reduced several important sources of heterogeneity that have complicated previous international studies, although they may also limit the generalizability of our findings to vaccinated populations or infections caused by later SARS-CoV-2 variants.

Overall, our study complements existing COVID-19 genetic studies by demonstrating that a systems biology framework can provide substantially greater biological interpretability than gene-level analyses alone, thereby facilitating future validation in larger Vietnamese and international cohorts.

### 4.9. Biological and Clinical Implications

Although this study was designed as an exploratory whole-exome sequencing investigation rather than a predictive genomic study, the findings provide several important biological and potential clinical implications. By integrating gene-based association analysis with pathway enrichment and interaction-network reconstruction, our results suggest that the exploratory findings may be more informatively interpreted at the level of biological pathways [32,33,34,80,81].

Rather than highlighting isolated candidate genes, our analyses consistently identified convergent biological modules involving RNA processing, mitochondrial bioenergetics, immunometabolism, transcriptional regulation, and membrane trafficking. These pathways have also emerged from transcriptomic, proteomic, and experimental studies of SARS-CoV-2 infection, supporting their biological relevance and prioritizing them for future functional investigation [35,37,38,39,62,71,74,82,83].

These findings also have implications for future precision medicine. Although host genetic information is not currently suitable for routine clinical risk prediction, integration of genomic variation with established clinical predictors—including age, comorbidities, inflammatory biomarkers, viral characteristics, and multi-omics data—may ultimately improve individualized risk stratification and therapeutic decision-making for severe viral infections. Such applications, however, will require validation in substantially larger, multi-center, and ethnically diverse cohorts [7,31,83,84].

Several pathways identified in this study, particularly those related to RNA metabolism, mitochondrial function, lipid homeostasis, and epigenetic regulation, are already being explored as potential therapeutic targets. While our study was not designed to evaluate therapeutic interventions, identifying these pathways in an independent Vietnamese cohort provides additional support for their continued investigation in translational research [38,55,57,62]. Finally, our study contributes valuable genomic data from the Vietnamese population, which remains substantially underrepresented in international COVID-19 genetic research. Increasing representation of diverse ancestral populations is essential for improving the generalizability of host-genetic discoveries, refining disease-risk prediction, and reducing disparities in precision medicine [31]. Nevertheless, the candidate genes, biological pathways, and functional modules identified in the present study should be regarded as hypothesis-generating rather than validated biomarkers or therapeutic targets. Independent replication, functional characterization, and prospective validation will be required before clinical translation can be considered.

### 4.10. Strengths and Limitations

One of the principal strengths of this study is the biological homogeneity of the cohort. All participants were of Vietnamese ancestry, infected with the SARS-CoV-2 Delta variant before vaccination, and classified using standardized national clinical criteria. These characteristics minimized important sources of heterogeneity related to ancestry, viral lineage, and vaccination status that have complicated many previous COVID-19 genetic studies. In addition, rigorous quality-control procedures and the absence of substantial inflation in the gene-based association statistics supports the absence of marked systematic inflation in the association test statistics.

A second strength is the integrative systems biology strategy adopted in this study. Rather than relying solely on variant-level associations, we combined whole-exome sequencing with gene-based association analysis, functional enrichment, interaction-network reconstruction, and module-based network analysis. The convergence of multiple complementary analytical approaches on the same biological processes—including RNA processing, mitochondrial bioenergetics, immunometabolism, transcriptional regulation, membrane trafficking, and cellular homeostasis—provides a complementary systems-level framework for organizing the exploratory findings.

Finally, this study contributes genomic data from the Vietnamese population, which remains underrepresented in international COVID-19 genetic studies. Increasing representation of diverse ancestral populations is essential for improving the generalizability of host genetic discoveries and reducing ancestry-related bias [7,31].

Several limitations should also be acknowledged. First, the relatively small sample size (*n* = 47 after QC) inherently constrained the statistical power to detect variants with modest effect sizes at exome-wide significance. Consequently, none of the individual candidate genes survived strict false discovery rate (FDR) correction. However, we used the nominal *p* < 0.01 threshold solely as an exploratory prioritization criterion for subsequent functional analyses.

Furthermore, the available medical records were limited in clinical detail. Height and weight were not consistently documented and therefore BMI could not be calculated reliably. Smoking status, detailed baseline biochemical or laboratory parameters, and specific inpatient treatment regimens were also incomplete or unavailable. These missing clinical variables could not be incorporated into the genetic association models; therefore, residual confounding by unmeasured or incompletely documented clinical factors cannot be excluded.

A key methodological limitation of the present study is the modest cohort size (*n* = 47; 24 mild/asymptomatic and 23 severe/critical participants), which substantially limits statistical power for single-variant association testing. Scenario-based post hoc power analysis showed that, at a nominal two-sided α = 0.05, estimated power ranged from 18.3% to 59.0% across the representative MAF and effect-size scenarios examined. Under the Bonferroni-adjusted significance threshold of α = 1.62 × 10^−7^ based on 308,014 valid single-variant tests, estimated power was ≤0.11% across the same scenarios (Supplementary Table S5). These results underscore the extremely limited ability of the present cohort to detect individual variant associations after stringent multiple-testing correction. Additionally, the primary multivariable logistic regression model included six covariates (age, sex, ethnicity, and PC1–PC3) in a genetic analysis set containing 23 severe/critical cases, resulting in a low events-per-variable ratio and an increased risk of model instability. The observed changes in nominal significance across reduced covariate models should therefore be interpreted cautiously, further underscoring the exploratory nature of the association signals.

Accordingly, the nominal *p* < 0.01 gene-level threshold was used solely as an exploratory prioritization criterion. None of the 44 prioritized genes remained significant after Benjamini–Hochberg correction, with adjusted *p* values of approximately 0.9994 (Supplementary Table S3). The prioritized genes and downstream enrichment and network analyses therefore should be interpreted as hypothesis-generating observations rather than statistically confirmed associations, independent validation, or causal mechanisms.

Second, age and sex were included as clinical covariates in the genetic association models, the significant age imbalance between the clinical severity groups remains an important consideration. The clinical characteristics summarized in Supplementary Table S1 describe the full enrolled cohort (*n* = 48), whereas the genetic association analyses were conducted in the 47 participants who passed genetic quality control. Statistical adjustment for age and sex reduces, but does not eliminate, the possibility of residual confounding, particularly given the limited sample size and the absence of comprehensive clinical covariates such as BMI, smoking status, detailed laboratory parameters, and treatment exposures. Specifically, despite incorporating age as a continuous covariate in our primary association models, the statistically significant age disparity between the mild/asymptomatic (median 39 years) and severe/critical (median 46 years) cohorts (*p* < 0.01) leaves room for potential residual biological confounding. Given that key biological pathways identified in our enrichment analysis—such as mitochondrial bioenergetics, epigenetic remodeling, and immunometabolic adaptation—are intimately interconnected with biological aging, we cannot completely rule out the possibility that a subset of candidate genes reflects age-associated cellular responses alongside SARS-CoV-2 susceptibility. Third, external replication and functional validation were beyond the scope of the present study. Consequently, the proposed biological modules and systems-level model represent biologically plausible hypotheses rather than experimentally validated mechanisms. Finally, because all participants were infected with the Delta variant before vaccination, the findings may not be directly generalizable to vaccinated populations or infections caused by subsequent SARS-CoV-2 variants. Future multicenter studies integrating larger cohorts, external replication, multi-omics approaches, and functional experiments will be required to validate the candidate pathways identified in the present study.

We acknowledge that tools like GeneMANIA and Metascape algorithmically force connections based on existing databases; therefore, the dense connectivity observed in our RNA processing and mitochondrial modules might partially reflect inherent database biases rather than exclusive SARS-CoV-2 pathogenesis signatures.

Overall, this study should be regarded as an exploratory, hypothesis-generating investigation that provides a systems-level framework for understanding host genetic susceptibility to severe COVID-19 in the Vietnamese population.

## 5. Conclusions

In this exploratory whole-exome sequencing study, we proposed a hypothesized biological framework of candidate genes and pathways potentially associated with COVID-19 severity in Vietnamese patients infected with the SARS-CoV-2 Delta variant. Rather than implicating a single susceptibility gene, our integrative analyses showed convergent exploratory patterns across six biological modules involving RNA processing, mitochondrial bioenergetics, metabolic reprogramming, transcriptional regulation, membrane trafficking, and cellular homeostasis.

By integrating gene-based association analysis with functional enrichment and interaction-network reconstruction, this study extends conventional variant-centered analyses toward a systems biology framework for interpreting host genetic variation. The convergence of these complementary analyses provides biologically coherent hypotheses and prioritizes functional pathways for future investigation.

Although confirmation in larger multicenter cohorts and experimental studies remains necessary, our findings provide exploratory genomic observations from an underrepresented Southeast Asian population and establish a foundation for future systems genetics, functional genomics, and precision medicine studies of COVID-19 and other emerging infectious diseases.

## Data Availability

Due to ethical and privacy restrictions regarding human genomic data, the raw whole-exome sequencing datasets generated during this study are not publicly available. De-identified, aggregate variant data and relevant clinical metadata that support the findings of this study are available from the corresponding author upon reasonable request, subject to approval by the Institutional Review Board.

## Supplementary Materials

The following supporting information can be downloaded at https://www.mdpi.com/article/10.3390/genes17080959/s1, Table S1: Baseline characteristics of the study participants according to COVID-19 severity; Table S2: Top 15 SNPs exhibiting suggestive association signals with COVID-19 severity (*p* < 0.01); Table S3: Forty-four genes with MAGMA gene-based association *p* < 0.01 selected for downstream functional analyses; Table S4: GeneMANIA interaction network statistics; Table S5: Scenario-based post hoc power analysis for single-variant association testing; Table S6. Sensitivity analysis of functional enrichment using an alternative gene-selection threshold; Figure S1: Bar chart showing interaction evidence (Co-expression (95.93%); Physical interaction (2.85%); Shared protein domains (1.22%); Figure S2: Comparison of single-variant effect estimates between the primary model and the age-, sex-, and ethnicity-adjusted model; Figure S3: Comparison of single-variant effect estimates between the primary model and the age- and sex-adjusted model.

## Abbreviations

The following abbreviations are used in this manuscript:

bp	base pair
Chr	chromosome
COVID-19	Coronavirus Disease 2019
EAF	Effect allele frequency
FDR	False discovery rate
GWAS	Genome-Wide Association Study
GO	Gene Ontology
GVCF	Genomic variant call format
KEGG	Kyoto Encyclopedia of Genes and Genomes
LD	Linkage disequilibrium
MAF	Minor allele frequency
MAGMA	Multi-marker Analysis of GenoMic Annotation
OR	Odds ratio
PCA	Principal component analysis
QC	Quality control
Q–Q	Quantile–Quantile
SARS-CoV-2	Severe Acute Respiratory Syndrome Coronavirus 2
SNP	Single-nucleotide polymorphism
WES	Whole-exome sequencing
WHO	World Health Organization
