# Supplementary material for: Whole-Exome Sequencing Explores Host Genetic Susceptibility of COVID-19 Severity in Unvaccinated Vietnamese Patients Infected with the Delta Variant: A Hypothesis-Generating Study
Source: Genes (Basel). 2026 Aug 15;17(8):959. doi: 10.3390/genes17080959 (PMC13511842; doi:10.3390/genes17080959)

SUPPLEMENTARY DATA

1. Supplementary Table S1: **Baseline characteristics of the study participants according to COVID-19 severity**

Variables	Mild/asymptomatic (n = 24)	Severe/critical (n = 24)	p-value
**Age (Median, IQR)**	39 (31–48)	46 (33–59)	**< 0.01**
**Sex (Male, %)**	12 (50.0%)	10 (41.7%)	0.56
**Comorbidities** (documented during hospitalization), **n (%)**	6 (25.0%)	2 (8.3%)	0.12

* **Abbreviations:** IQR, Interquartile Range.

2. Supplementary Table S2: Top 15 SNPs exhibiting suggestive association signals with COVID-19 severity ($p < 0.01$)

SNP	Chr	Position (bp)	Effect Allele	EAF	OR	Nominal p-value
rs3846449	4	100,259,126	C	0.296	13.012	0.001
rs12639833	4	100,267,372	T	0.295	12.843	0.001
rs2009181	4	100,262,547	G	0.297	13.045	0.001
rs2241894	4	100,266,133	C	0.266	10.935	0.001
rs2173201	4	100,250,970	A	0.298	13.080	0.001
rs3734381	6	118,887,303	C	0.266	14.723	0.001
rs13146	3	124,462,808	T	0.149	0.034	0.002
rs10764176	10	18,242,311	G	0.160	0.044	0.002
rs12487906	3	124,468,453	C	0.146	0.030	0.002
rs12497383	3	124,468,494	T	0.146	0.030	0.002
rs12497347	3	124,468,393	T	0.147	0.031	0.002
rs1801019	3	124,456,742	C	0.147	0.031	0.002
rs4678149	3	124,462,058	G	0.147	0.031	0.002
rs9875527	3	124,462,259	T	0.147	0.031	0.002
rs2242247	3	124,463,656	T	0.147	0.031	0.002

Abbreviations: Chr, chromosome; bp, base pair; EAF, effect allele frequency; OR, odds ratio; SNP, single-nucleotide polymorphism.

3. Supplementary Table S3 : Forty-four genes with MAGMA gene-based association P < 0.01 selected for downstream functional analyses.

No.	Gene	Chr	Genomic position (bp)	Mapped SNPs	Z-statistic	p-value	Adjusted p-value (Benjamini Hochberg)
1	BUD31	7	99,001,243 – 99,018,739	5	29.248	0.0017	0.999361769
2	SREBF1	17	17,713,163 – 17,745,331	1	28.466	0.0022	0.999361769
3	PDAP1	7	98,990,796 – 99,011,305	4	27.308	0.0032	0.999361769
4	MC3R	20	54,818,788 – 54,826,371	1	27.241	0.0032	0.999361769
5	FAM200A	7	99,142,423 – 99,161,115	10	26.871	0.0036	0.999361769
6	ATP5MF	7	99,054,284 – 99,068,824	15	26.867	0.0036	0.999361769
7	TRAPPC6B	14	39,615,515 – 39,644,634	16	26.823	0.0037	0.999361769
8	CTXN3	5	126,979,713 – 126,995,822	1	26.717	0.0038	0.999361769
9	FPGT	1	74,658,896 – 74,676,033	2	26.682	0.0038	0.999361769
10	OR2F1	7	143,652,020 – 143,659,608	21	26.633	0.0039	0.999361769
11	ZNF655	7	99,151,034 – 99,175,576	14	26.533	0.0040	0.999361769
12	MGARP	4	140,185,817 – 140,206,492	1	26.515	0.0040	0.999361769
13	FGFBP3	10	93,664,845 – 93,674,258	1	26.461	0.0041	0.999361769
14	UMPS	3	124,444,213 – 124,469,620	58	26.459	0.0041	0.999361769
15	ATP5MF-PTCD1	7	99,012,862 – 99,068,824	56	26.454	0.0041	0.999361769
16	GET4	7	911,191 – 937,573	41	26.295	0.0043	0.999361769
17	LHX9	1	197,876,635 – 197,906,106	2	26.285	0.0043	0.999361769
18	CPSF4	7	99,031,563 – 99,056,496	35	26.192	0.0044	0.999361769
19	PNN	14	39,639,387 – 39,653,922	17	26,101	0.0045	0.999361769
20	PTCD1	7	99,012,862 – 99,050,811	38	25,959	0.0047	0.999361769
21	MRPS26	20	3,021,675 – 3,030,396	14	25,924	0.0048	0.999361769
22	CEP85L	6	118780435- 119036238	3	25,572	0.0052	0.999361769
23	GPN1	2	27846515 - 27875213	5	25,561	0.0052	0.999361769
24	CCDC121	2	27846908 - 27856898	4	25,292	0.0057	0.999361769
25	MRPS34	16	1820391 - 1828152	17	25,223	0.0058	0.999361769
26	GNRH2	20	3019268 - 3027891	13	25,217	0.0058	0.999361769
27	EME2	16	1818223 - 1828128	21	24,982	0.0062	0.999361769
28	PROX2	14	75318236 - 75335562	5	24,684	0.0067	0.999361769
29	GID4	17	17937611 - 17973219	35	2,466	0.0068	0.999361769
30	YLPM1	14	75225025 - 75305513	4	24,454	0.0072	0.999361769
31	CALHM2	10	105205043 - 105217162	4	2,441	0.0073	0.999361769
32	ZNF470	19	57073401 - 57101465	40	24,382	0.0073	0.999361769
33	NME3	16	1818821 - 1826743	17	24,189	0.0077	0.999361769
34	PKNOX1	21	44389643 - 44455188	1	24,159	0.0078	0.999361769
35	ZFP28	19	57045317 - 57069670	20	24,148	0.0078	0.999361769
36	SUN1	7	850194 - 916057	158	24,113	0.0079	0.999361769
37	HDAC1	1	32752708 - 32800727	1	23,911	0.0083	0.999361769
38	SNRPD3	22	24946618 - 24972432	1	23,733	0.0088	0.999361769
39	NUP160	11	47798170 - 47875057	33	23,706	0.0088	0.999361769
40	SERPINB11	18	61365194 - 61392623	65	23,636	0.0090	0.999361769
41	CCRL2	3	46443721 - 46455988	10	23,535	0.0092	0.999361769
42	PHF14	7	11008495 - 11210750	2	23,514	0.0093	0.999361769
43	VPS29	12	110927830 - 110944916	2	23,465	0.0094	0.999361769
44	TACR1	2	75272090 - 75431645	2	23,326	0.0098	0.999361769

4. Supplementary Table S4: GeneMANIA interaction network statistics

Gene	Degree	Betweenness	Closeness Centrality	Clustering Coefficient	Topological Coefficient	Major functional module
CPSF4	168	0.051555735	0.661538	0.437229	0.305195	RNA processing & mRNA maturation
MRPS34	135	0.067866268	0.651515	0.442105	0.310465	Mitochondrial function
NME3	115	0.011241811	0.558442	0.6	0.387387	Mitochondrial function
BUD31	102	0.011119436	0.581081	0.604396	0.374564	RNA processing & mRNA splicing
PDAP1	89	0.00811879	0.565789	0.641026	0.401923	RNA processing / transcription
ATP5MF	83	0.070214542	0.623188	0.411765	0.321477	Mitochondrial oxidative phosphorylation
TRAPPC6B	83	0.034612357	0.589041	0.391667	0.276563	Membrane trafficking
SNRPD3	77	0.012032891	0.544304	0.564103	0.388773	RNA processing & spliceosome
FPGT	74	0.039960806	0.597222	0.275	0.275915	Cellular metabolism
FAM200A	71	0.020196526	0.558442	0.439394	0.360417	Cellular homeostasis
HDAC1	67	0.105623341	0.68254	0.338462	0.302885	Epigenetic regulation
GPN1	63	0.046015712	0.623188	0.403509	0.331579	RNA processing / transcription
MRPS26	58	0.002496608	0.518072	0.777778	0.447222	Mitochondrial translation
ZNF470	57	0.02155173	0.551282	0.305556	0.291005	Transcriptional regulation
ZFP28	55	0.005451886	0.5	0.535714	0.342857	Transcriptional regulation
CCDC121	53	0.023907702	0.558442	0.363636	0.2949	Nuclear organization
PHF14	53	0.020697803	0.551282	0.397436	0.346154	Chromatin regulation
EME2	52	0.004898491	0.551282	0.711111	0.382927	DNA repair / nuclear regulation
ZNF655	50	0.025966358	0.565789	0.450549	0.358974	Transcriptional regulation
PTCD1	50	0.050623946	0.544304	0.530303	0.367117	Mitochondrial RNA metabolism
PNN	50	0.004047229	0.5	0.555556	0.428571	RNA processing & nuclear organization
UMPS	39	0.017237426	0.5375	0.6	0.421053	Nucleotide metabolism
GNRH2	32	0.030057601	0.518072	0.25	0.24024	Cell communication
SUN1	31	0.001639816	0.488636	0.6	0.457143	Nuclear envelope organization
NUP160	31	0.015350673	0.551282	0.424242	0.333333	Nuclear pore / RNA export
GET4	27	0.011837625	0.551282	0.527273	0.356818	Protein targeting
CALHM2	24	0.015184028	0.551282	0.525641	0.37247	Calcium signaling
MC3R	23	0.020912124	0.467391	0.25	0.28125	Immunometabolism
FGFBP3	23	0.051923133	0.573333	0.19697	0.294872	Growth factor signaling / metabolism

Gene	Degree	Betweenness	Closeness Centrality	Clustering Coefficient	Topological Coefficient	Major functional module
VPS29	23	0.004010736	0.494253	0.527778	0.447712	Endosomal trafficking
SERPINB11	22	0.025601906	0.494253	0.25	0.25	Cellular stress response
SREBF1	22	0.006627464	0.518072	0.694444	0.423423	Lipid metabolism
TACR1	21	0.008272199	0.483146	0.333333	0.285714	Neuroimmune signaling
OR2F1	20	0.018043517	0.505882	0.285714	0.28125	Cell signaling
YLPM1	19	0.009769898	0.518072	0.392857	0.405405	RNA processing / transcription
PKNOX1	17	5.43E-04	0.472527	0.8	0.446078	Transcription factor
MGARP	17	0.022711367	0.5	0.277778	0.287582	Mitochondrial dynamics
GID4	15	0.002617197	0.505882	0.607143	0.461806	Protein degradation
LHX9	13	6.29E-04	0.380531	0.666667	0.5	Transcription factor
CTXN3	13	0.007943145	0.43	0.333333	0.362319	Cell communication
PROX2	12	0.010575691	0.483146	0.333333	0.3125	Transcriptional regulation
CEP85L	10	0.012841218	0.52439	0.142857	0.329365	Cell-cycle organization
CCRL2	8	0.012100461	0.511905	0.2	0.311404	Immune cell trafficking

5. Supplementary Table S5: Scenario-based post-hoc power analysis for single-variant association testing

Scenario	MAF	OR	Power at α=0.05	Power at Bonferroni $\alpha=1.62\times10^{-7}$
1	0.05	2.5	18.30%	~0%*
2	0.1	2.5	29.56%	0.01%
3	0.2	2.5	45.02%	0.03%
4	0.2	3	58.97%	0.11%

* QUANTO reported a power value of 0.0000 for this scenario; the value is therefore reported as approximately 0%.

Power was estimated using QUANTO 1.2.4 for an unmatched case-control design with 23 severe/critical cases and 24 mild/asymptomatic controls, assuming a log-additive genetic model, a two-sided test, and a population risk (Kp) of 0.20. The nominal significance level was α = 0.05. The Bonferroni-adjusted significance threshold was calculated as $\alpha = 0.05/308{,}014 = 1.62 \times 10^{-7}$, based on the number of variants yielding valid regression P-values in the primary single-variant analysis.

6. Supplementary Table S6. Sensitivity analysis of functional enrichment using an alternative gene-selection threshold.

Functional theme / Pathway	Pathway / Term ID	P<0.01 (44 genes)	P<0.05 (368 genes)	Interpretation
**Pre-mRNA processing & RNA splicing** *(Hubs: CPSF4, BUD31)*	R-HSA-72203 / GO:0008380	**5 genes** ($P = 6.31 \times 10^{-5}$, $Log_{10}(P) = -4.20$) • *Genes:* **CPSF4**, **BUD31**, *SNRPD2*, *SRSF1*, *NUP160*	**15 genes** ($P = 1.29 \times 10^{-5}$, $Log_{10}(P) = -4.89$) • *Genes:* **CPSF4**, **BUD31**, *PRPF8*, *SF3B1*, *SRSF2*, *SNRPD2*, *SRSF1*, *NUP160*, *HNRNPA1*, +6 genes	Preserved (Enhanced)

**Functional theme / Pathway**	**Pathway / Term ID**	**P<0.01 (44 genes)**	**P<0.05 (368 genes)**	**Interpretation**
**Mitochondrial ribosome, Energy metabolism & ATP synthesis** *(Hubs: MRPS34, NME3, ATP5MF)*	GO:0005739 / R-HSA-1428517	Enriched (P = 3.16 x 10^{-3}, Log_{10}(P) = -2.50) • *Genes:* **MRPS34**, **NME3**, **ATP5MF**, *NDUFA4*	Enriched (P = 1.58 x 10^{-4}, Log_{10}(P) = -3.80) • *Genes:* **MRPS34**, **NME3**, **ATP5MF**, *NDUFA4*, *MRPL12*, *COX5B*, *UQCRB*, +5 genes	Preserved
**Chromatin remodeling & Epigenetic regulation** *(Hub: HDAC1)*	GO:0006325 / R-HSA-3214815	Enriched (P = 5.01 x 10^{-3}, Log_{10}(P) = -2.30) • *Genes:* **HDAC1**, *RBBP4*, *SMARCA4*	Enriched (P = 8.12 x 10^{-4}, Log_{10}(P) = -3.09) • *Genes:* **HDAC1**, *HDAC2*, *RBBP4*, *SMARCA4*, *KAT2B*, *EP300*, +3 genes	Preserved
**ER-to-Golgi transport & Intracellular trafficking** *(Hub: TRAPPC6B)*	R-HSA-199991 / GO:0006888	Enriched (P = 7.94 x 10-3, Log_{10}(P) = -2.10) • *Genes:* **TRAPPC6B**, *GOPC*, *COPB1*	Enriched (P = 1.10 x 10^{-3}, Log_{10}(P) = -2.96) • *Genes:* **TRAPPC6B**, *TRAPPC3*, *GOPC*, *COPB1*, *SEC23A*, *SAR1A*, +2 genes	Preserved
**Metabolic regulation & Cellular homeostasis** *(Hub: PDAP1)*	GO:0019222 / GO:0008152	Enriched (P = 8.91 x 10^{-3}, Log_{10}(P) = -2.05) • *Genes:* **PDAP1**, *PGAM1*, *ENO1*	Enriched (P = 2.45 x 10^{-3}, Log_{10}(P) = -2.61) • *Genes:* **PDAP1**, *PGAM1*, *ENO1*, *PKM*, *LDHA*, +4 genes	Preserved
**Immunologic signatures & Immune cell responses**	MSigDB: M4691, M8142, M3144, M7565	Enriched (P < 0.01 across top immune modules) • *Genes: CD14*, *FCGR1A*, *TNF*, *IL1B*, *CCR1*	Enriched (P < 0.001 broad immune signature network) • *Genes: CD14*, *FCGR1A*, *TNF*, *IL1B*, *CCR1*, *CXCR4*, *TLR4*, +18 genes	Preserved
**GPCR signaling / Class A/1 Rhodopsin-like receptors**	R-HSA-373076	**4 genes** (P = 1.38 x 10^{-3}, Log $_{10}$(P) = -2.86) • *Genes: GPR183*, *CCR1*, *CXCR4*, *FPR2*	**11 genes** (P = 7.59 x 10^{-4}, Log $_{10}$(P) = -3.12) • *Genes: GPR183*, *CCR1*, *CXCR4*, *FPR2*, *LPAR1*, *P2RY12*, +5 genes	Preserved

7. Supplementary Figure S1: Bar chart showing interaction evidence (Co-expression (95.93%); Physical interaction (2.85%); Shared protein domains (1.22%)

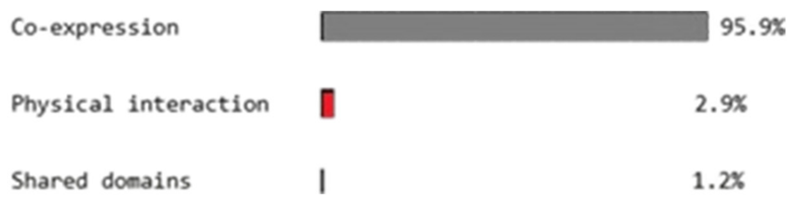

8. Supplementary Figure S2: Comparison of single-variant effect estimates between the primary model and the age-, sex-, and ethnicity-adjusted model.

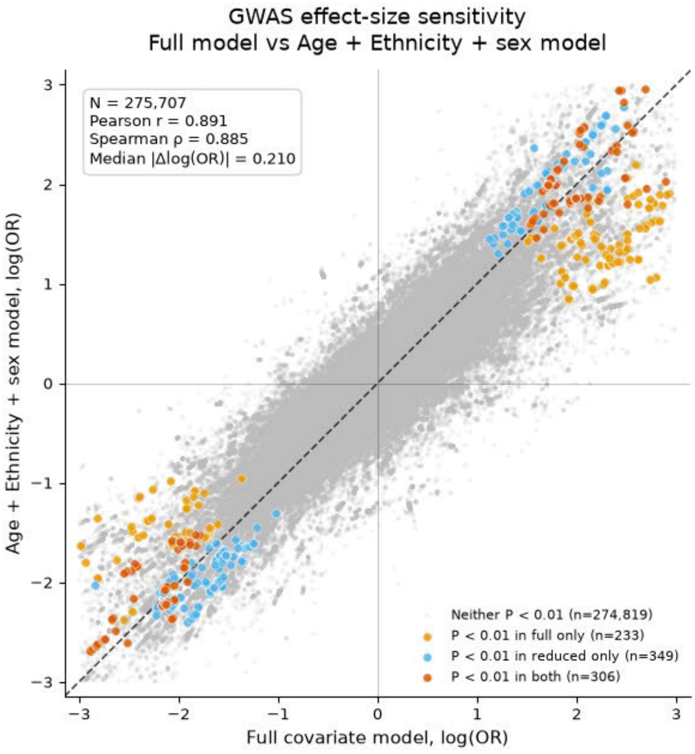

9. Supplementary Figure S3: Comparison of single-variant effect estimates between the primary model and the age- and sex-adjusted model.

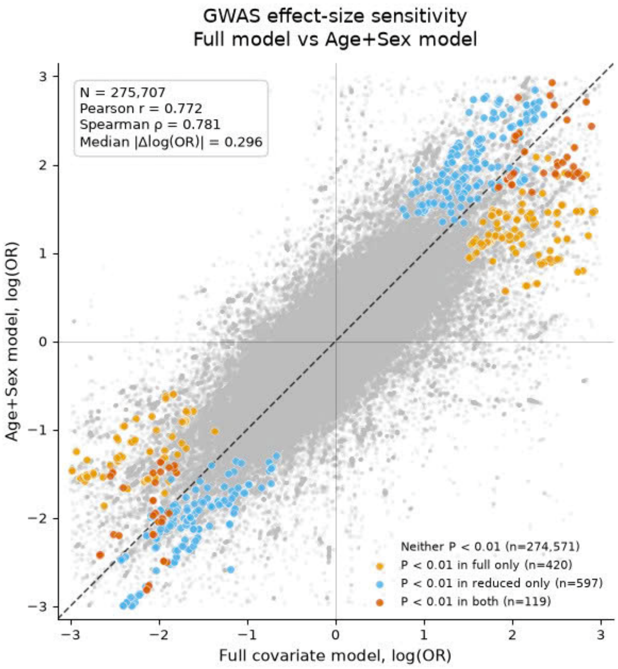

Supplement: Supplementary file 1 [file genes-17-00959-s001.zip › genes-4476447-supplementary.pdf]
